# Regulation of TAK1/TAB1-Mediated IL-1β Signaling by Cytoplasmic PPARβ/δ

**DOI:** 10.1371/journal.pone.0063011

**Published:** 2013-04-30

**Authors:** Josefine Stockert, Alexander Wolf, Kerstin Kaddatz, Evelyn Schnitzer, Florian Finkernagel, Wolfgang Meissner, Sabine Müller-Brüsselbach, Michael Kracht, Rolf Müller

**Affiliations:** 1 Institute of Molecular Biology and Tumor Research (IMT), Philipps University, Marburg, Germany; 2 Rudolf Buchheim Institute for Pharmacology, Giessen, Germany; University of Geneva, Switzerland

## Abstract

The peroxisome proliferator-activated receptor subtypes PPARα, PPARβ/δ, PPARγ are members of the steroid hormone receptor superfamily with well-established functions in transcriptional regulation. Here, we describe an unexpected cytoplasmic function of PPARβ/δ. Silencing of PPARβ/δ expression interferes with the expression of a large subset of interleukin-1β (IL-1β)-induced target genes in HeLa cells, which is preceded by an inhibition of the IL-1β-induced phosphorylation of TAK1 and its downstream effectors, including the NFκBα inhibitor IκBα (NFKBIA) and the NFκBα subunit p65 (RELA). PPARβ/δ enhances the interaction between TAK1 and the small heat-shock protein HSP27, a known positive modulator of TAK1-mediated IL-1β signaling. Consistent with these findings, PPARβ/δ physically interacts with both the endogenous cytoplasmic TAK1/TAB1 complex and HSP27, and PPARβ/δ overexpression increases the TAK1-induced transcriptional activity of NFκB. These observations suggest that PPARβ/δ plays a role in the assembly of a cytoplasmic multi-protein complex containing TAK1, TAB1, HSP27 and PPARβ/δ, and thereby participates in the NFκB response to IL-1β.

## Introduction

Peroxisome proliferator-activated receptors (PPARs) are nuclear receptors that function as ligand-inducible transcription factors [1], [2]. Consistent with their regulation by fatty acids and eicosanoid metabolites, PPARs function as modulators of lipid metabolism and inflammatory responses. The three PPAR subtypes (α, β/δ and γ) activate their target genes through binding to PPAR response elements (PPREs) as heterodimers with members of the retinoid X receptor (RXR) family. Genome-wide analyses have identified PPRE-mediated repression as a major mechanism of transcriptional regulation by unliganded PPARβ/δ, and revealed that a subset of these repressed genes is activated by an agonist-mediated switch [3].

There is a large body of evidence implicating PPARβ/δ in inflammation-associated processes. This evidence is based on the observation that the expression of PPARβ/δ or its ligands is regulated by different cytokines (such as TNFα, TGFβ or IL-4) or small molecular modulators of inflammation, such as leukotrienes and hydroxyeicosatetraenoic acid [4]–[8]. Furthermore, PPARβ/δ can modulate the outcome of cytokine-triggered signaling transduction, for example by TGFβ [9]. Multiple molecular mechanisms underlying these observations have been identified, which places PPARβ/δ into a complex regulatory network.

In addition to the canonical PPRE-mediated mechanism, PPARβ/δ can regulate genes without making direct DNA contacts by directly interacting with specific transcription factors, although the molecular mechanisms involved are poorly understood. For example, PPARβ/δ interacts with the p65 subunit of the NFκB dimer, and PPARβ/δ ligands have been described to modulate NFκB signaling by unknown mechanisms [10]–[13]. Furthermore, PPARβ/δ has been reported to interact with BCL6 in macrophages in the absence of ligand, which prevents the repression of inflammatory genes by BCL6 [14]. Deletion of *Ppard* or application of a PPARβ/δ ligand abolishes the sequestration of BCL6, resulting in the repression of BCL6 target genes. This PPARβ/δ – BCL6 interaction is involved in atherogenesis-associated inflammation [14]. Thus, deletion of PPARβ/δ in foam cells was atheroprotective through the increased availability of the inflammatory repressor BCL6 and the downregulation of pro-inflammatory genes, including *Mcp1, IL-1b* and *MMP9*.

Polarization of macrophages toward the anti-inflammatory M2a state is PPARβ/δ-dependent in adipose tissue and liver [6], [7]. Adipocytes and hepatocytes locally produce IL-4 and IL-13, thereby establishing a reciprocal functional crosstalk between parenchymal cells and resident macrophages. IL-4 or IL-13 exposure leads to PPARβ/δ activation in macrophages, possibly through direct transcriptional induction and/or a cytokine-induced endogenous ligand [6], [7]. Consistent with the idea that PPARβ/δ promotes the anti-inflammatory polarization of immune cells is the observation that PPARβ/δ agonists inhibit Th1 and Th17 responses in a mouse model of experimental allergic encephalomyelitis [15].

Several lines of evidence point to a negative regulatory role for PPARβ/δ in inflammatory responses of the skin. Thus, mice deficient for PPARβ/δ showed an increased inflammatory response to the topical application of O-tetradecanoylphorbol-13-acetate [16]. Furthermore, PPARβ/δ stimulates the production of the secreted IL-1 receptor antagonist in dermal fibroblasts, which dampens the inflammatory response [17]. On the other hand, PPARβ/δ is overexpressed in skin lesions in the majority of psoriasis patients concomitant with a global gene expression profile reminiscent of a PPARβ/δ signature [12], [18].

IL-1β is a cytokine with a prominent role in promoting inflammation [19], [20]. A central player in the transduction of IL-1β signals is the MAP3K TAK1 (TGFβ-activated protein kinase 1) [21]. TAK1 activation requires sequential phosphorylation of its catalytic domain and K63-linked polyubiquitination by the E3 ligase TRAF6. Polyubiquitination of TAK1 depends on its interaction with TAB (TAK1-binding protein) adaptor proteins 1–3, which recruit K63-linked ubiquitinated TRAF6 to TAK1. The assembled TAK1/TAB complex determines the output signal and mediates activation of NFκB or the MAP2Ks MKK4/7 and MKK3/6. TAK1-dependent NFκB activation involves phosphorylation-dependent (IKK) K48-linked ubiquitination and subsequent degradation of IκBα and K63-linked ubiquitination of the regulatory IKK subunit NEMO (NFκB essential modulator). Perturbation of signal integration by TAK1–TAB or IKK–NEMO complexes can result in inflammation and cancer. Downstream of TAK1–TAB and IKK, the NFκB subunit p65 integrates signals at the molecular level. Nuclear activity of p65 is regulated by multiple phosphorylations, polyubiquitination and acetylation, which impinges on the co-ordinated recruitment of transcription factors and co-activators in concert with chromatin-regulatory mechanisms at target gene promoters [22], [23]. However, the efficient IL-1β triggered upregulation of secondary inflammatory mediators (e.g. IL-6, IL-8, cyclooxygenase 2) also requires the simultaneous activation of both transcriptional and post-transcriptional pathways. IL-1β target gene responses thus require stabilization of mRNAs and translational derepression by signal-mediated processes through the p38–MK2-pathway. However, the regulation by post-transcriptional mechanisms and post-translational modifications and their interplay with transcriptional pathways are only partly understood.

Despite numerous findings pointing to an essential role for PPARβ/δ in modulating inflammatory responses, its role in IL-1β signal transduction remains elusive. The same applies to the other two PPAR subtypes, PPARα and PPARγ. In the present study, we addressed this question for PPARβ/δ by a combination of genome-wide approaches and biochemical technologies. As a model we chose in HeLa cells, since these cells show a strong transcriptional response to IL-1β and represent a well established cell system for the analysis of IL-1β signaling [24]–[30]. The data obtained from these studies provide strong evidence for a functional interaction of PPARβ/δ with the TAK1– NFκB signaling axis. This role for PPARβ/δ is supported by our finding that not only TAK1, but also the small heat shock protein HSP27 can interact with PPARβ/δ. Previous studies by others have identified HSP27 as a modulator of IL-1β signal transduction [24], although its function in inflammatory signaling is still not entirely clear. It has been implicated in the regulation of TRAF6 ubiquitination, IKK activation and IκB degradation [31]–[33] and specifically in feedback regulation of TAK1-dependent pathways. Similar functions for other small HSPs, which mainly function as molecular chaperones, are not known.

## Materials and Methods

### Cell Culture and Cytokines

HeLa and WI-38 cells were obtained from the ATCC, HEK293T cells from Open Biosystems (TLA-HEK293T). HCT116-PPARD^+/+^ and HCT116-PPARD^−/−^ cells [34] were kindly provided by K.W. Kinzler. HEK293T, HEK293IL-1R cells (HEK293T cells stably expressing the IL-1 receptor) [35], HeLa and HCT116 cells were cultured in Dulbecco’s modified Eagle’s medium (DMEM), complemented with 10% fetal calf serum, 2 mM L-glutamine, 100 U/ml penicillin, 100 µg/ml streptomycin. Cells were maintained in a humidified incubator at 37°C and 5% CO_2_. Recombinant human IL1β was purchased from Thermo Scientific.

### Antibodies

Neutralizing monoclonal antibody against human IL-6 was purchased from R&D Systems and normal mouse IgG from Santa Cruz Biotechnology (sc-2025). Antibodies against the following proteins or peptides were used for immunoblotting and immunoprecipitation: actin (JLA20; EMD) and TAK1 (sc-7162), TAB1 (sc-13956), p65 NF-kB (sc-372), P(S536)-p65 (sc-3033), IkBa (sc-9242), P(S32)-IkBa (sc-2859) all from Santa Cruz, MYC (9E10), HA (12CA5), GFP (clone 7.1 and 13.1) all from Roche, FLAG M2 (F1804, Sigma), P(T180/Y182)-p38 MAPK (36–850, Invitrogen), and p38 MAPK (raised against ISFVPPPLDQEEMES; rat p38a with C-terminal 15 residues) [36], TAK1 (4505), P(T187)-TAK1 (4536), TAB1 (C25E9) all from Cell signaling, HSP27 (ADI-SPA-803) from Stressgen, and GFP-Trap_A coupled to agarose beads from Chromotek.

### Cell Lysis and Analysis of Proteins

Cells were lysed in (50 mM TrisHCl, pH 7.5, 100 mM NaCl, 0,1 mM EGTA, 1 mM EDTA, 1% Triton X-100, 50 mM NaF, 1 µM Microcystin, 1 mM Na_3_VO_4,_ 5 mM sodium pyrophosphate, 0,1% ß-mercaptoethanol and a Roche protease inhibitor mix). Cell lysates were subjected to SDS-PAGE on 8–12,5% gels and immunoblotting was performed as described [6]. HeLa cells transfected for reporter gene assays were lysed in ß-galactosidase lysis buffer as described [6].

For immunoprecipitation of PPARβ/δ, cell extracts were incubated with Protein G Sepharose 4 Fast Flow coupled to 1 µg of FLAG antibodies for 2 h with gentle rocking at 4°C. Beads were then washed two times with lysis buffer (0,5 M NaCl) and once with washing buffer. Beads were boiled for 5 min in 2× Roti-Load (Roth) before loading on SDS-PAGE.

For immunoprecipitation of GFP-TAK1 or GFP-TAB1, cell extracts were incubated with GFP-Trap_A antibodies, coupled to agarose beads for 2 h with gentle rocking at 4°C. Beads were then washed two times with lysis buffer (0,5 M NaCl) and once with washing buffer. Beads were boiled for 5 min in 2x Roti-Load (Roth) before loading on SDS-PAGE.

Celluar fractionation was performed with the Qproteome Cell Compartment kit according to the manufacturer’s manual (Qiagen, Hilden, Germany).

IL-6 levels in cell culture medium was determined with a commercial IL-6 ELISA kit (RayBiotech, Inc.) according to the manufacturer’s manual.

### Plasmids, Transfections, Reporter Gene Assays

3xFLAG-PPARβ/δ was generated by cloning the coding sequence of mPPARβ/δ N-terminally fused to a triple FLAG tag [37] into pcDNA3.1 (+) zeo (Invitrogen, Karlsruhe, Germany). 3xFLAG-PPARβ/δ 4-165 was created using site-directed mutagenesis (Stratagene) and 3xFLAG-PPARβ/δ 166–440 was amplified from 3xFLAG-PPARβ/δ sequence by PCR using a 5′ primer containing BamHI site and a 3′ primer containing XhoI site. The PCR fragment was ligated into BamHI and XhoI sites of 3xFLAG-PPARβ/δ-pcDNA3.1. Primers are listed in Table S1. pE-CFP-PPARβ/δ was a kind gift of B. Desvergne.

GFP-TAK1 1–579 (wt), 1–493, 1–362 and 1–296 were generated by amplification of full length TAK1 and C-terminal truncated mutants using 5 different anti-sense primers. These constructs were cloned into pCDNA3.1/NT-GFP-TOPO vector (Invitrogen) resulting in GFP-tagged TAK1 constructs.

Expression vectors for pCMV-HA-TAK1, pCMV-HA-TAK1K63W, pSV40-ß-Galactosidase [38], pE-GFP-TAB1 [39], pE-GFP-TAK1, pCS2MT-MYC-TAB1, pCDNA3-HA-TAB1 [35], and p-NF-kB(3)luc-promotor [40] have been described. pcDNA3-HA-Hsp27 [41] was a kind gift of M. Gaestel.

Calcium phosphate transfections and reporter gene assays were performed as described [42].

### siRNA Interference

siRNA transfections were carried out essentially as described [3] using pools of 4 siRNAs for genes (Dharmacon and Qiagen). Cells were seeded at a density of 5×10^5^ cells per 6 cm dish in 4 ml DMEM with 10% FCS and cultured for 2 h. 1280 ng siRNA in 100 µl OptiMEM (Invitrogen) and 20 µl HiPerfect (Qiagen, Hilden, Germany) were mixed and incubated for 5–10 min at room temperature prior to transfection. The cells were replated 24 h post-transfection at a density of 5×10^5^ cells per 6 cm dish. Transfection was repeated 48 h after start of the experiment, and cells were passaged after another 24 h. Twenty-four hours following the last transfection, cells were incubated in serum-free medium overnight. Cells were stimulated and harvested after 1, 2, 3, 6 and 8 hrs. siRNA sequences are listed in Table S2.

### Quantitative RT-PCR

cDNA was synthesized from 0.1–1 µg of RNA using oligo(dT) and random primers and the iScript kit (Biorad, Germany). qPCR was performed in a Mx3000P Real-Time PCR system (Stratagene, La Jolla, CA) for 40 cycles at an annealing temperature of 60°C. PCR reactions were carried out using the Absolute QPCR SYBR Green Mix (Abgene, Hamburg, Germany) and a primer concentration of 0.2 µM following the manufacturer’s instructions. *L27* was used as normalizer. Comparative expression analyses were statistically analyzed by Student’s *t*-test (two-tailed, equal variance) and corrected for multiple hypothesis testing via the Bonferroni method. RT-qPCR primer sequences are listed in Table S3.

### Microarrays

Human Agilent 4-plex Array 44K were used for the analysis of the gene expression of the different samples in a reference-design assay as previously published [43]. Raw microarray data were normalized using the ‘loess’ method implemented within the marray package of R/Bioconductor [44]. Probes were assigned to genes as described [3] using Ensembl release 67. Hybridizations from two biological replicates per conditions were performed in a flip-color reference design. Probes were considered regulated if they had a minimum intensity value of 5, a Benjamini-Hochberg corrected t-test based p-value of <0.05 and a comparison specific change as specified in the Results. Raw and normalized microarray data from this publication have been submitted to the EBI ArrayExpress and assigned the identifier [accession: E-MTAB-1212]. All data is MIAME compliant.

### ChIP-qPCR

ChIP-qPCR was performed and evaluated as described [43] using the following antibodies: IgG pool (I5006; Sigma-Aldrich, Steinheim, Germany); α-PPARβ/δ (sc-7197), α-RXRα (sc-774), α-p65 (sc-372). Primer sequences are listed in Table S4.

## Results

### PPARβ/δ Depletion Attenuates IL-1β Induction of a Subset of Target Genes in HeLa Cells

**Figure 1:**
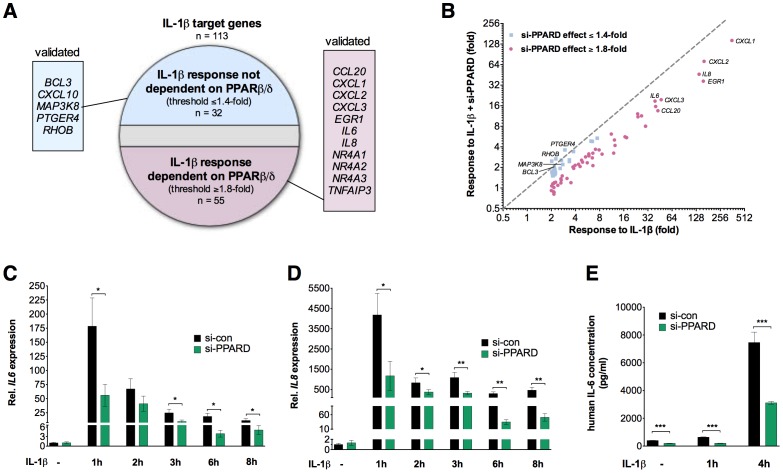
Effect of PPARβ/δ depletion on global transcriptional response to IL-1β. (**A**) Diagrammatic representation of IL-1β target genes (threshold ≥2-fold; *n* = 113) showing a reduced induction by IL-1β (threshold ≥1.8-fold; *n* = 55) or no significant effect on induction (threshold ≤1.4-fold; *n* = 32) after PPARβ/δ depletion. HeLa cells were treated with control siRNA *(*si-con) or *PPARD*-directed siRNA (si-PPARD) followed by IL-1β (10 ng/ml) for 1 h (see Figure S1 for knockdown efficiency). Expression patterns were determined by microarray analyses and genes showing a ≥2-fold regulation were identified (Datasets S1 and S2). The observed regulation was verified by RT-qPCR, as exemplified for the genes listed in the boxed areas and shown in Figures S2 and S3. (**B**) Scatter plot showing the IL-1β response of individual genes with or without *PPARD* silencing (microarray data from panel A). The dashed line shows the ideal position of genes theoretically unaffected by si-PPARD. Blue data points: effect ≤1.4-fold; red data points: effect ≥1.8-fold. (**C**), (**D**) Effect of PPARβ/δ depletion on the time course of the IL-1β-mediated induction of the *IL6* (C) and *IL8* (D) gene determined by RT-qPCR. (**E**) Effect of PPARβ/δ depletion on IL-1β-induced IL-6 secretion in HeLa cells determined by ELISA (1 h and 4 h stimulation with IL-1β). Values represent averages ±SD (*n* = 3). ***, **, *significant difference between si-con and si-PPARD-treated cells (*p*<0.001, *p*<0.01, *p*<0.05 by t-test).

To identify potential functional interactions between the IL-1β and PPARβ/δ signaling pathways we performed microarray analyses of HeLa cells treated with IL-1β (10 ng/ml) in the presence of a siRNA targeting *PPARD* (si-PPARD; Figure S1) or a control siRNA (si-con). As illustrated by the diagram in Figure 1A, 113 genes were regulated by IL-1β (≥2-fold change). A subset of 55 of these genes (48.7%) showed a clearly reduced IL-1β response (threshold≥1.8-fold) in PPARβ/δ-depleted cells (Figure 1B; Datasets S1 and S2), including pivotal IL-1β target genes like *IL6* and *IL8*, while the induction by IL-1β of another 32 genes (28.3%) was not significantly affected (threshold ≤1.4-fold; Figure S3). Twenty-six IL-1β target genes were not categorized due to a borderline effect of si-PPARD (1.4- to 1.8-fold). This categorization was verified by RT-qPCR for a large number of genes from both groups (Figures 1C, D; Figures S2, S3). The effect of PPARβ/δ depletion on *IL6* induction was also detectable at the protein level (IL-6 secretion; Figure 1E).

**Figure 2:**
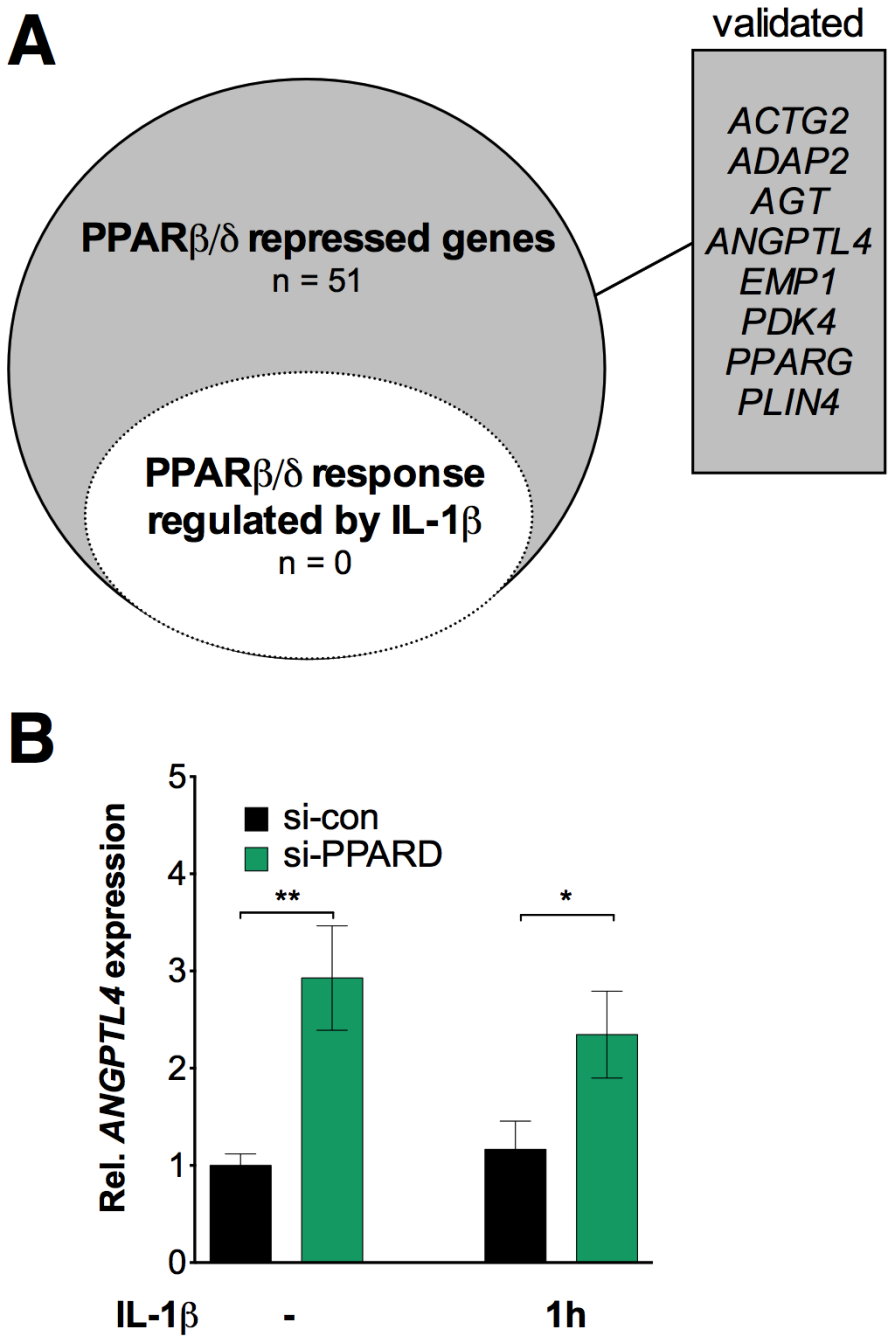
Effect of IL-1β on PPARβ/δ target genes. (**A**) Set (*n* = 51) of PPARβ/δ target genes (defined as genes upregulated by siRNA-mediated PPARβ/δ depletion) and empty subset of these genes affected by IL-1β (*n* = 0). HeLa cells were treated and analyzed as in Figure 1 (threshold ≥1.8-fold regulation; Dataset S3). Verified genes are listed in the boxed area and shown in Figure S4. (**B**) Effect of IL-1β on the PPARβ/δ response of the *ANGPTL4* gene. Values represent averages ±SD (*n* = 3). ***, **, *significant difference between si-con and si-PPARD-treated cells (*p*<0.001, *p*<0.01, *p*<0.05 by t-test).

We also asked whether, conversely, IL-1β would affect the transcription of PPARβ/δ target genes. Toward this end, we identified all genes derepressed by si-PPARD in HeLa cells by transcriptional profiling (≥1.8-fold change; n = 51) and analyzed whether this response was altered by IL-1β. As shown in Figure 2A and Dataset S3 this was not the case for any PPARβ/δ target gene, including classical PPAR target genes, such as *ANGPTL4* (Figures 2B and S4). Taken together, these observations demonstrate that the crosstalk between IL-1β and PPARβ/δ is unidirectional, as it specifically affects IL-1β signaling.

We also found that this function of PPARβ/δ is not regulated by ligands, since neither the PPARβ/δ agonist GW501516 [45] nor the inhibitory inverse agonist ST247 [46] had any significant effect on IL-1β-mediated target gene induction (Figure S5). In view of these findings it is important to note that the siRNA-mediated inhibitory effect was not only observed with the pool of four *Ppard*-targeting siRNAs used throughout this study, but also with three individual siRNAs from this pool (Figure S6).

### PPARβ/δ Modulates IL-1β-mediated IL-6 Signaling

**Figure 3:**
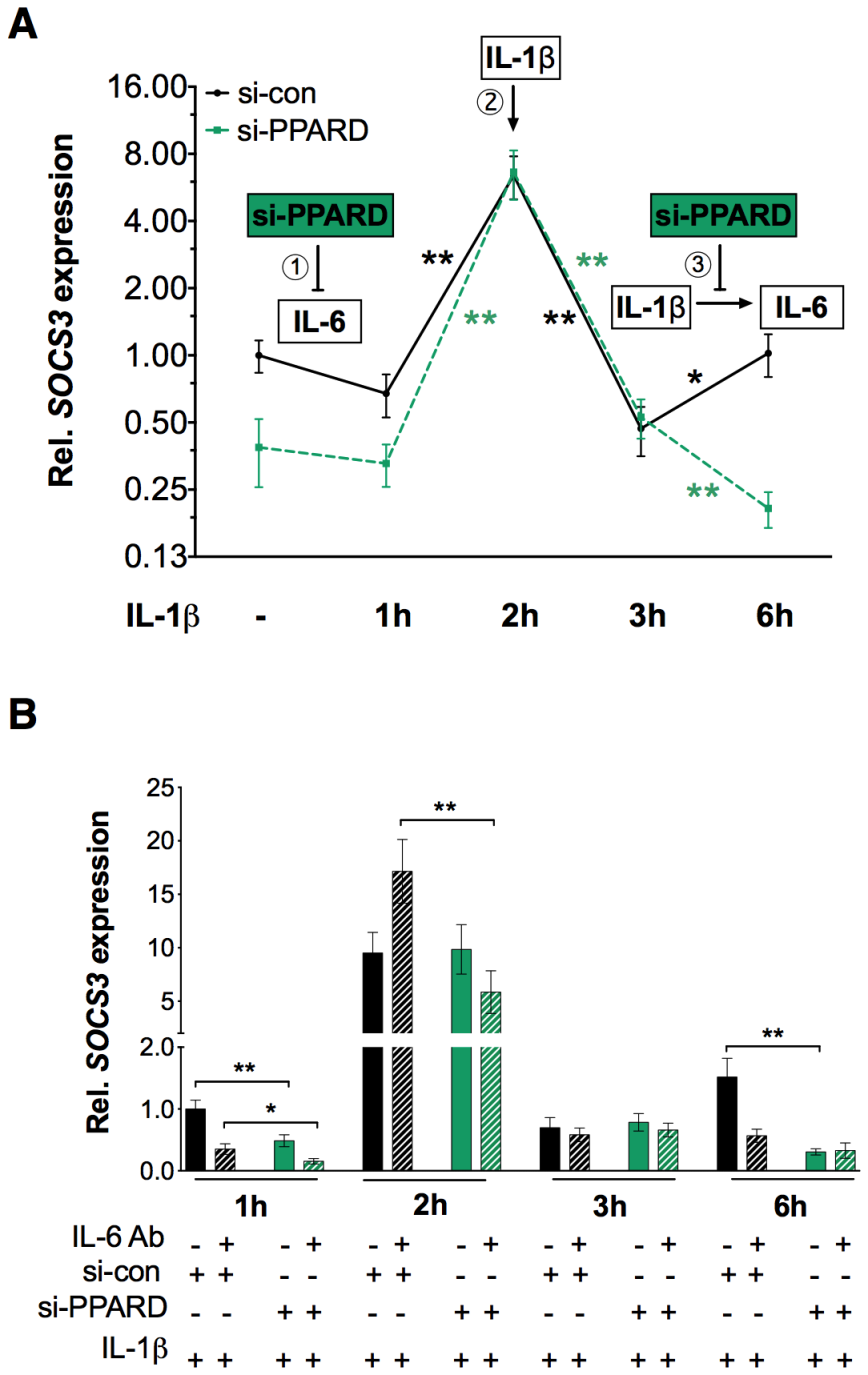
Modulation of IL-1β-mediated IL-6/SOCS3 signaling by PPARβ/δ. (**A**) Time course of *SOCS3* mRNA expression in IL-1β (10 ng/ml) stimulated HeLa cells in the presence of si-con and si-PPARD. Three regulatory events are recognizable and indicated by numbers: (1) IL-1β independent down-regulation of *SOCS3* by si-PPARD, presumably resulting from PPARβ/δ-regulated basal IL-6 expression; (2) direct *SOCS3* induction by IL-1β; and (3) upregulation of *SOCS3* as a consequence of IL-1β induced IL-6 secretion, which is inhibited by siPPARD. **, *significant difference between time points (*p*<0.01, *p*<0.05 by t-test). (**B**) The same experimental setup as in panel A, except that neutralizing antibodies against IL-6 or control IgG was included in the cell culture medium, starting 15 h before IL-1β stimulation. **, *significant difference between si-con and si-PPARD-treated cells (*p*<0.01, *p*<0.05 by t-test).

We next addressed the question whether PPARβ/δ might also regulate a feed-forward loop constituted by IL-1β and its target gene *IL6*. A feed-forward loop, a three-gene pattern, is composed of two input factors, one of which regulates the other, both jointly regulating a target gene [47]. As shown by the black line in Figure 3A, IL-1β induced the known IL-6 target gene *SOCS3* in a complex manner. A reproducible (albeit statistically not significant) initial decrease (phase 1) preceded a strong temporary induction at 2 h (phase 2), followed by another rise in expression between 3 and 6 h (phase 3). PPARβ/δ depletion led to clearly decreased initial *SOCS3* expression (phase 1) and prevented the late induction during phase 3, but had no effect on the peak levels in phase 2. To separate direct IL-1β effects on *SOCS3* from secondary effects mediated by IL-1β-induced *IL6* we performed the same experiment in the presence or absence of neutralizing IL-6 antibodies. The data in Figure 3B clearly show that *SOCS3* expression during phase 1 and 3 was dependent on IL-6, while its peak induction at 2 h was not. These observations suggest that phase 1 expression is partially due to basal level of IL-6 expression, phase 2 represents a direct induction by IL-1β, and phase 3 results from IL-1β-induced IL-6 secretion. These data assign PPARβ/δ a positive regulatory function in an IL-1β/IL-6-mediated feed-forward loop, which increases basal level expression of their common target gene *SOCS3* and extends its induction by IL-1β.

### PPARβ/δ Modulates p65 Interaction at Co-regulated Target Genes

**Figure 4:**
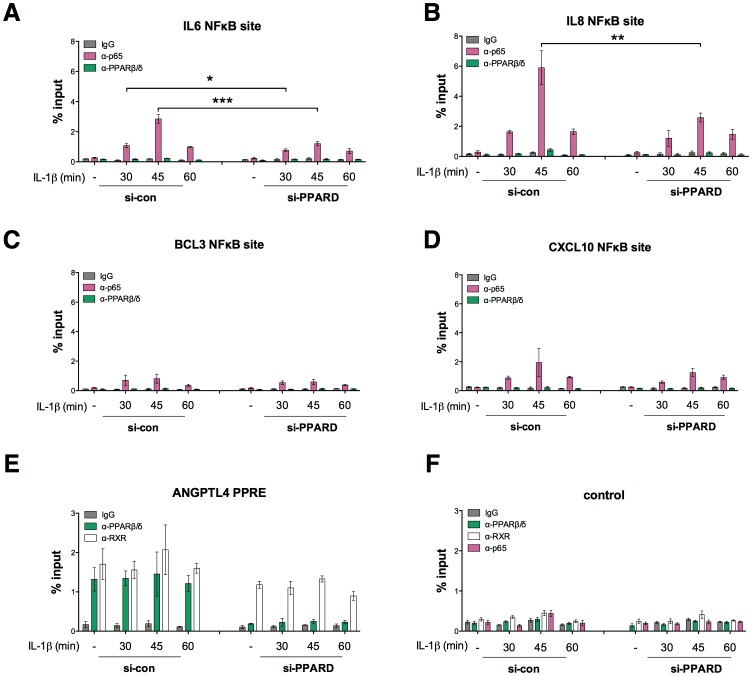
Modulation of p65 binding to NFκB target genes *in vivo* by PPARβ/δ. HeLa cells were treated with IL-1β and siRNAs as indicated and ChIP assays were performed with antibodies against PPARβ/δ (green), RXR (white) or p65 (red) or control IgG (grey). PCR primers were designed to detect the NFκB binding sites of the *IL6* (**A**), *IL8* (**B**), *BCL3* (**C**) and *CXCL10* (**D**) genes, the triple-PPRE of the ANGPTL4 gene (**E**) or an irrelevant genomic control region (**F**). Relative amounts of amplified DNA in immunoprecipitates were calculated by comparison with 1% of input DNA. Results are expressed as % input and represent averages of triplicates (± S.D). ***, **, *significant differences between si-con and si-PPARD-treated cells (*p*<0.001, *p*<0.01, *p*<0.05 by t-test).

Most of the IL-1β-regulated genes identified above are proven or potential NFκB target genes. We therefore performed chromatin immunoprecipitation (ChIP) analyses to investigate whether the PPARβ/δ effect on IL-1β-induced transcription might be linked to NFκB site occupancy. Consistent with the expression data (Figure 1C, D) we observed a clear inhibition of p65 binding to the *IL6* and *IL8* genes 30–45 min after IL-1β stimulation (Figure 4A, B), whereas no significant effect was seen on p65 recruitment to the *BCL3* and *CXCL10* genes (Figure 4C, D). Furthermore, in agreement with the expression data in Figure 2, we did not observe any difference on the recruitment of PPARβ/δ or its obligatory dimerization partner RXR to their target gene *ANGPTL4* upon IL-1β stimulation (Figure 4E). Finally, no significant binding of the p65, PPARβ/δ and RXR antibodies to an irrelevant genomic control region was observed (Figure 4F).

### PPARβ/δ Modulates TAK1-mediated Signaling to NFκB

**Figure 5:**
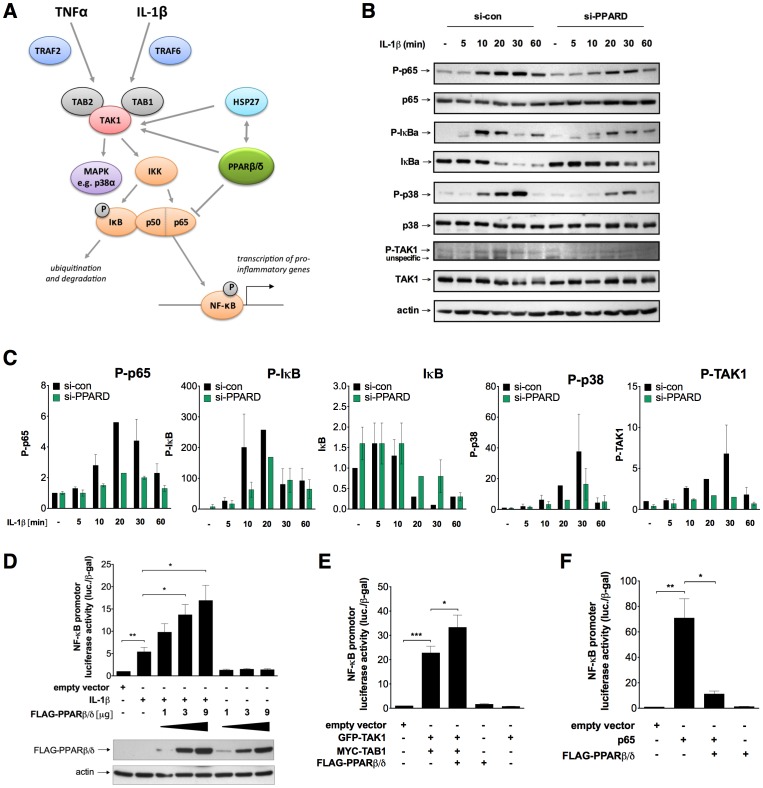
Modulation of TAK1-mediated signaling to NFκB by PPARβ/δ. (**A**) Components of cytokine-induced TAK1 signaling and effects of PPARβ/δ identified in the present study. (**B**) Immunoblot analysis of the indicated proteins at different time points after IL-1β stimulation of si-con and si-PPARD treated HeLa cells. P-p65: p65 phosphorylated at serine-536, P-IκB: serine 32; P-p38: threonine-180 and tyrosine-182; P-TAK1: threonine-187. The siRNA effect on PPARβ/δ protein levels in this experiment is shown in Figure S1. (**C**) Quantification of data obtained by immunoblotting for phosphorylated p65, IκB, p38 and TAK1 as in panel B. Values for phosphoproteins were normalized to signals measured for total protein levels. (**D**) Effect of PPARβ/δ overexpression on IL-1β induced NFκB activity. HeLa cells were transfected with a NFκB-luciferase reporter plasmid and FLAG-PPARβ/δ expression vector or empty vector, and treated with IL-1β for 4 h as indicated. Luciferase activities were determined in cell lysates and normalized to β-galactosidase expressed from a cotransfected CMV-lacZ plasmid. (**E**) Effect of PPARβ/δ overexpression on TAK1/TAB1-induced NFκB activity. Experimental setup as in panel D, expect that TAK1 and TAB1 expression vectors were used instead of IL-1β. (**F**) Effect of PPARβ/δ overexpression on p65-induced NFκB activity. Experimental setup as in panel D, expect that a 65 expression vector was used instead of IL-1β.

We next addressed the question as to whether PPARβ/δ impinges on specific steps of the canonical IL-1β signaling pathway, which activates the transcription factor NFκB via the TRAF6– TAK1/TAB1/2– IKK – IκB – NFκB cascade (Figure 5A). We therefore investigated the effect of *PPARD* silencing on the expression and phosphorylation status of several key components of this pathway (Figure 5B, C). This analysis revealed in PPARβ/δ-depleted cells a decreased phosphorylation of the NFκB subunit p65 at serine-536, which represents an activating modification mediated by multiple protein kinases, including IKKs [22], [40]. Consistent with this finding we observed a decreased phosphorylation of IκB at serine-32, which marks IκB for ubiquitin-mediated degradation, concomitantly with a delayed degradation of IκB (Figure 5B, C). The simultaneous inhibition of p38 phosphorylation at threonine-180/tyrosine-182 suggests that PPARβ/δ exerts its modulatory effect upstream of IKKs (see Figure 5A). This notion is in agreement with the observed decrease in phosphorylation of TAK1 at threonine-187 (Figure 5B, C). PPARβ/δ depletion also inhibited the TNFα-induced transcription of common TNFα and IL-1β target genes (Figure S7), suggesting that the TAK1/TAB complex is targeted by PPARβ/δ as a point of convergence of the TNFα – TRAF2 and IL-1β – TRAF6 pathways (see Figure 5A).

These conclusions based on siRNA interference are supported by a gain-of-function approach analyzing the effect of PPARβ/δ overexpression in transient luciferase reporter gene assays measuring NFκB activity. Both, the IL-1β-triggered activation of NFκB (Figure 5D) and the TAK1/TAB1-induced NFκB activation (Figure 5E) were enhanced by the co-expression of PPARβ/δ in a dose-dependent manner. In contrast, PPARβ/δ inhibited p65-induced NFκB activation (Figure 5F), which is consistent with the proposed inhibitory effect of PPARβ/δ ligands on NFκB activity (see Introduction). In contrast, no significant effect was seen on basal level of NFκB activity in all three experiments, suggesting that the stimulatory PPARβ/δ effect is dependent on IL-1β induced TAK1/TAB1 signaling.

### PPARβ/δ Interacts with Cytoplasmic TAK1 and TAB1

**Figure 6:**
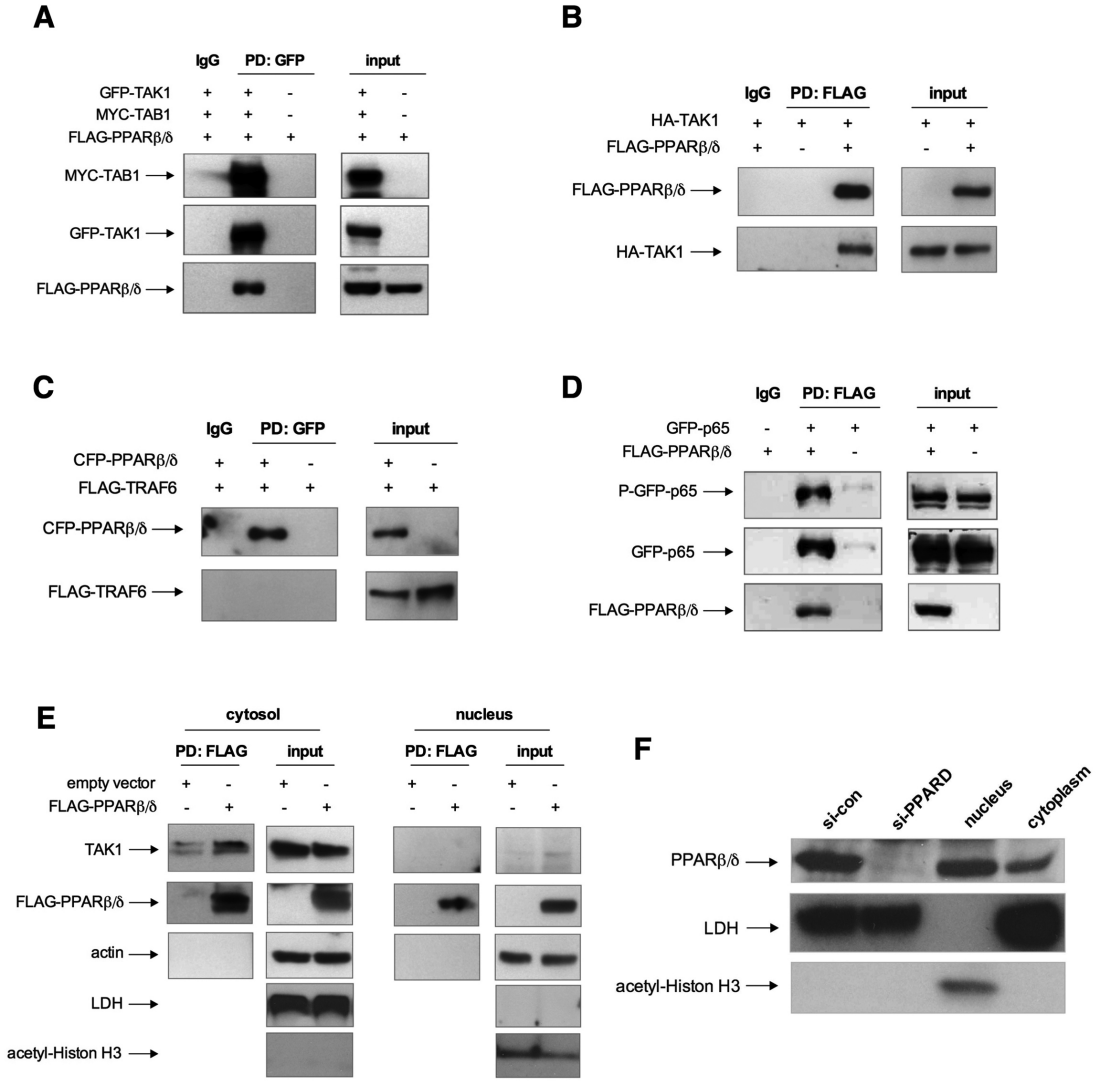
Complex formation of PPARβ/δ with TAK1/TAB1. (**A**) Co-immunoprecipitation of PPARβ/δ with TAK1 and TAB1. HEK293IL-1R cells were transfected with expression vectors for MYC-tagged TAB1, GFP-tagged TAK1 and FLAG-tagged PPARβ/δ. Pulldown (PD) was carried out with an antibody against GFP, and immunoblotting with antibodies against TAB1, TAK1 or FLAG. Input lanes were loaded with 50 µg protein (3% of the amount used for IPs). (**B**) Co-immunoprecipitation of PPARβ/δ and TAK1. HEK293IL-1R cells were transfected with expression vectors for HA-tagged TAK1 and/or FLAG-tagged PPARβ/δ. Pulldown (PD) was carried out with an antibody against FLAG, and immunoblotting with antibodies against TAK1 or FLAG. (**C**) Co-immunoprecipitation of PPARβ/δ and TRAF6. HEK293IL-1R cells were transfected with expression vectors for FLAG-tagged TRAF6 and/or CFP-tagged PPARβ/δ. Pulldown (PD) was carried out with an antibody against GFP, and immunoblotting with antibodies against GFP or TRAF6. (**D**) Co-immunoprecipitation of PPARβ/δ and p65. HEK293IL-1R cells were transfected with expression vectors for FLAG-tagged PPARβ/δ and/or HA-tagged p65. Pulldown (PD) was carried out with an antibody against FLAG, and immunoblotting with antibodies against FLAG or HA. (**E**) Cytoplasmic interactions of FLAG-PPARβ/δ with endogenous TAK1. HEK293T cells were transfected with expression vectors for FLAG-tagged PPARβ/δ. Cells were fractionated into cytoplasmic and nuclear fractions, pulldown (PD) was carried out with an antibody against FLAG, and immunoblotting with antibodies against TAK1, FLAG and actin. (**F**) Subcellular localization of endogenous PPARβ/δ in HEK293T cells. Cytoplasmic and nuclear fractions were isolated and analyzed by immunoblotting with an antibody against PPARβ/δ. Antibodies against lactate dehydrogenase (LDH) and acetyl-Histon H3 were included in panels E and F to control for the purity of the cytoplasmic and nuclear fractions.

We explored the presumptive effect of PPARβ/δ further by co-immunoprecipitation studies using HEK293T cells, which are particularly well suited for the efficient expression of exogenous proteins and therefore represent the most widely used experimental system for this purpose. Co-expression of MYC-tagged TAB1, GFP-tagged TAK1 and FLAG-tagged PPARβ/δ resulted in the immunoprecipitation of TAK1 complexed with both TAB1 and PPARβ/δ (Figure 6A). Interaction of FLAG-PPARβ/δ with HA-TAK1 was also observed in the absence of MYC-TAB1 (Figure 6B). In agreement with our assumption that the regulatory effect by PPARβ/δ is exerted down-stream of TRAF6, we did not detect any interaction of the two proteins upon overexpression of FLAG-tagged TRAF6 and CFP-tagged PPARβ/δ (Figure 6C). Using the same experimental setup we also reproduced the described interaction of PPARβ/δ and p65 (Fig. 6D). Cell fractionation studies showed that FLAG-PPARβ/δ forms complexes with endogenous TAK1 predominantly in the cytoplasm (Figure 6E). Consistent with this finding, a substantial fraction of PPARβ/δ was localized to the cytoplasm in HEK293T cells (Figure 6F).

### Identification of PPARβ/δ and TAK1 Domains Involved in their Physical and Functional Interactions

**Figure 7:**
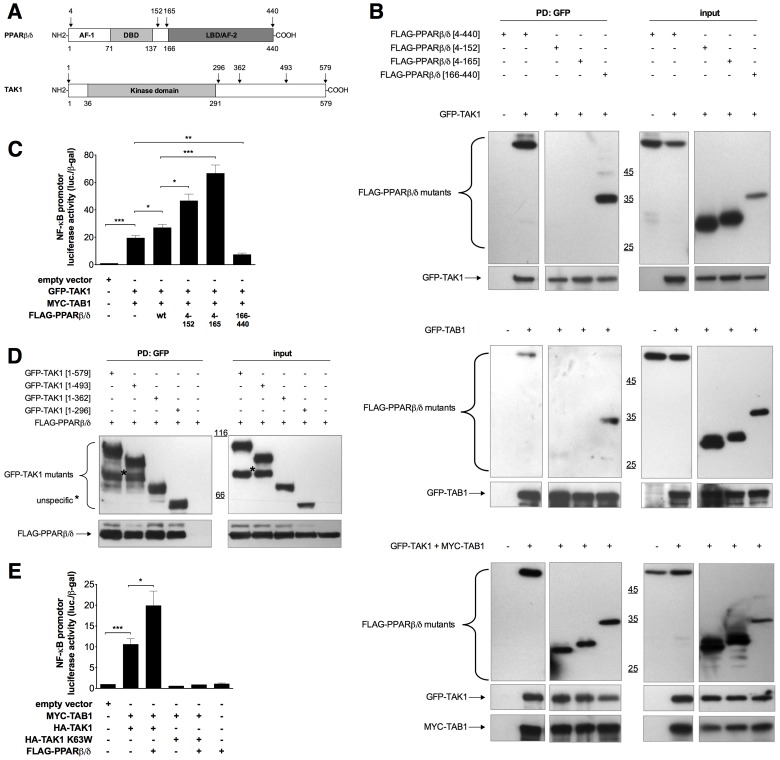
Identification of PPARβ/δ and TAK1 domains involved in their physical and functional interactions. (**A**) Domain structure of PPARβ/δ and TAK1. Deletion mutants were constructed as to preserve or remove specific domains in PPARβ/δ (N-terminal activation domain AF1, DNA-binding domain DBD, ligand binding activation domain LBD/AF2 or the hinge region between the DBD and the LBD/AF2 domains) and in TAK1 (kinase domain). Additional truncations at the C-terminus of TAK-1 mimic known splice variants [65]. (**B**) Interaction of PPARβ/δ deletion mutants with TAK1 and TAB1. Experimental details were as in Figure 6A. (**C**) Effect of PPARβ/δ deletion mutants on TAK1/TAB1 induced NFκB activity. Experimental details were as in Figure 5E. (**D**) Interaction of TAK1 deletion mutants with PPARβ/δ. Experimental details were as in Figure 6A. (**E**) Dependence of the PPARβ/δ enhancement of NFκB activity on catalytically active TAK1. Values represent averages ±SD (*n* = 3–5). ***, **, *significant differences between samples as indicated (*p*<0.001, *p*<0.01, *p*<0.05 by t-test).

We next sought to delineate the domains in TAK1 and PPARβ/δ involved in complex formation and the PPARβ/δ-mediated regulation of NFκB activity. For this purpose, we constructed a range of deletion mutants (Figure 7A) and tested these in co-immunoprecipitation and NFκB reporter gene assays. As shown in Figure 7B, wild-type PPARβ/δ and the C-terminal fragment 166–440 interacted with either GFP-TAK1 or GFP-TAB1, whereas the 4–152 and 4–165 fragments did not. Co-expression of GFP-TAK1 and MYC-TAB1, however, resulted in complex formation of both proteins with all three FLAG-PPARβ/δ fragments. These data indicate that two different domains of PPARβ/δ are involved with TAK1/TAB1 interaction. While the C-terminal domain interacts with both TAK1 and TAB1 individually, the N-terminal portion of PPARβ/δ appears to interact selectively with TAK1/TAB1 complexes. A functional correlation was established by a luciferase reporter gene assay performed in the presence of co-expressed MYC-TAB1 and GFP-TAK1 (Figure 7C). The results of this assay define the N-terminal portion as the functionally important region of PPARβ/δ, and assign a negative regulatory role to the C-terminus. This is suggested by the increased activity of the C-terminally truncated PPARβ/δ fragments 4–152 and 4–165 and the repressive effect of the C-terminal fragment 166–440.

Analysis of TAK1 deletion mutants showed that truncations of TAK1 starting at positions 296, 362, 493 or 579 had no detectable effect, indicating that the sequences located C-terminally to the catalytic domain are dispensable for its interaction with PPARβ/δ (Figure 7D). This conclusion is consistent with the observation that the stimulatory function of PPARβ/δ on NFκB activation is dependent on the catalytic activity of TAK1, since the catalytically inactive mutant K63W was unable to mediate the PPARβ/δ effect (bars 4 and 5 in Figure 7E).

### PPARβ/δ Interacts with Cytoplasmic HSP27

**Figure 8:**
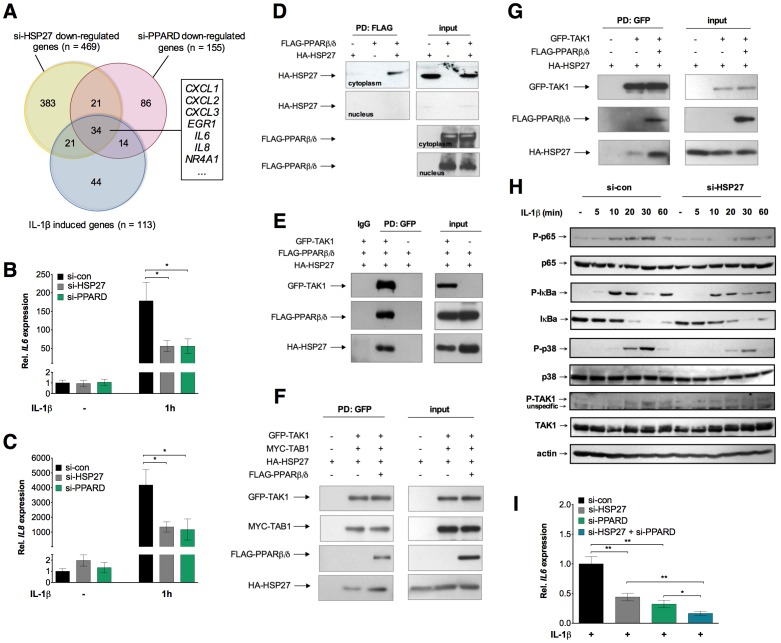
Functional and physical interaction of PPARβ/δ with HSP27. (**A**) Venn Diagram showing the overlaps of genes induced by IL-1β (blue; threshold ≥2-fold; *n* = 34; *n* = 113), downregulated by si-HSP27 (yellow; threshold ≥1.5-fold; *n* = 469) or downregulated by si-PPARD (red; threshold ≥1.8-fold; *n* = 155). HeLa cells were treated with control siRNA *(*si-con) or gene-specific siRNAs followed by IL-1β (10 ng/ml) for 1 h (see Figure S1 and S8 for knockdown efficiency). Expression patterns were determined by microarray analyses and genes showing a ≥1.5-fold regulation were identified (Dataset S4). The observed regulation was verified by RT-qPCR, as exemplified for the genes listed in the boxed areas and shown in Figure S9. (**B, C**) Effect of HSP27 or PPARβ/δ depletion on the IL-1β-mediated induction of the *IL6* (B) and *IL8* (C) genes in HeLa cells determined by RT-qPCR. Values represent averages ±SD (*n* = 3). ***, **, *significant difference between si-con and si-PPARD-treated cells (p<0.001, *p*<0.01, *p*<0.05 by t-test). (**D**) Cytoplasmic interaction of PPARβ/δ and HSP27 detected by co-immunoprecipitation. HEK293T cells were transfected with expression vectors for HA-tagged HSP27 and/or FLAG-tagged PPARβ/δ. Cells were fractionated into cytoplasmic and nuclear fractions, pulldown (PD) was carried out with an antibody against FLAG and immunoblotting with anti-HA antibodies. (**E**) Immunoprecipitation of GFP-tagged TAK1 in complexes with FLAG-tagged PPARβ/δ, HA-tagged HSP27. HEK293IL-1R cells were transfected with the indicated expression vectors. Pulldown (PD) was carried out with an antibody against GFP, and immunoblotting with antibodies against TAK1, FLAG or HA. (**F**) Co-expression of PPARβ/δ enhances the interaction of HSP27 and TAK1. HEK293IL-1R cells were transfected with expression vectors for GFP-tagged TAK1, MYC-tagged TAB1, HA-tagged HSP27 and FLAG-tagged PPARβ/δ. Pulldown (PD) was carried out with an antibody against GFP, and immunoblotting with antibodies against TAK1, TAB1, FLAG and HSP27. (**G**) Same experiment as in panel F, except that MYC-TAB1 was omitted. (**H**) Immunoblot analysis of the indicated proteins at different time points after IL-1β stimulation of si-con and si-HSP27 treated HeLa cells. Details as in Figure 5B. The siRNA effect on HSP27 protein levels in this experiment is shown in Figure S8. (**I**) Effects of siRNA-mediated depletion of PPARβ/δ or/and HSP27 on the IL-1β-induced transcription of *IL6* (6 h stimulation with IL-1β). Values represent averages ±SD (*n* = 3). ***, **, *significant differences (*p*<0.001, *p*<0.01, *p*<0.05 by t-test).

As the small heat shock protein HSP27 has previously been reported to enhance the TAK1-mediated activation of NFκB [24], [31]–[33], we investigated a potential interplay of TAK1, HSP27 and PPARβ/δ. Microarray analyses identified a total of 113 IL-1β target genes, 469 genes down-regulated by HSP27-siRNA and 155 genes down-regulated by PPARβ/δ depletion (Figure 8A; see Figure S8 for a characterization of si-HSP27). Intriguingly, a large group of 34 IL-1β target genes was co-regulated by both HSP27 and PPARβ/δ, including *IL6* and *IL8* (Figure 8B, C; Dataset S4; Figure S9).

We also identified HSP27 in a yeast two-hybrid screen as a new potential interaction partner of PPARβ/δ. This finding was confirmed by the co-immunoprecipitation experiment in Figure 8D, which shows a clear interaction of HA-tagged HSP27 and FLAG-tagged PPARβ/δ in HEK293T cells. This interaction was detectable only in the cytoplasm. It has previously been shown that the N-terminal HA-tag does not interfere with the function of HSP27 [41], [48]. Co-expression of HA-HSP27, GFP-TAK1 and FLAG-PPARβ/δ resulted in the immunoprecipitation of TAK1 in complexes with both HSP27 and PPARβ/δ (Figure 8E). The data also indicate that the co-expression of PPARβ/δ enhances the interaction of HSP27 and TAK1 both in the presence (Figure 8F) or absence (Figure 8G) of co-expressed MYC-TAB1.

These interactions of PPARβ/δ and HSP27 seem to have similar consequences, since HSP27 depletion had comparable effects on TAK1 - NFκB signaling components (Figure 8H) as si-PPARD (Figure 5B), i.e., decreasing p65, IκBα, p38 and TAK1 phosphorylation. The inhibitory effect of the siRNA-mediated knockdown of PPARβ/δ or HSP27 on *IL6* expression was ∼50% in both cases, and additive when both genes were silenced simultaneously (Figure 8I). PPARβ/δ and HSP27 thus have functionally similar effects on TAK1, suggesting that both proteins cooperate with TAK1 to maximize signaling to NFκB.

**Figure 9:**
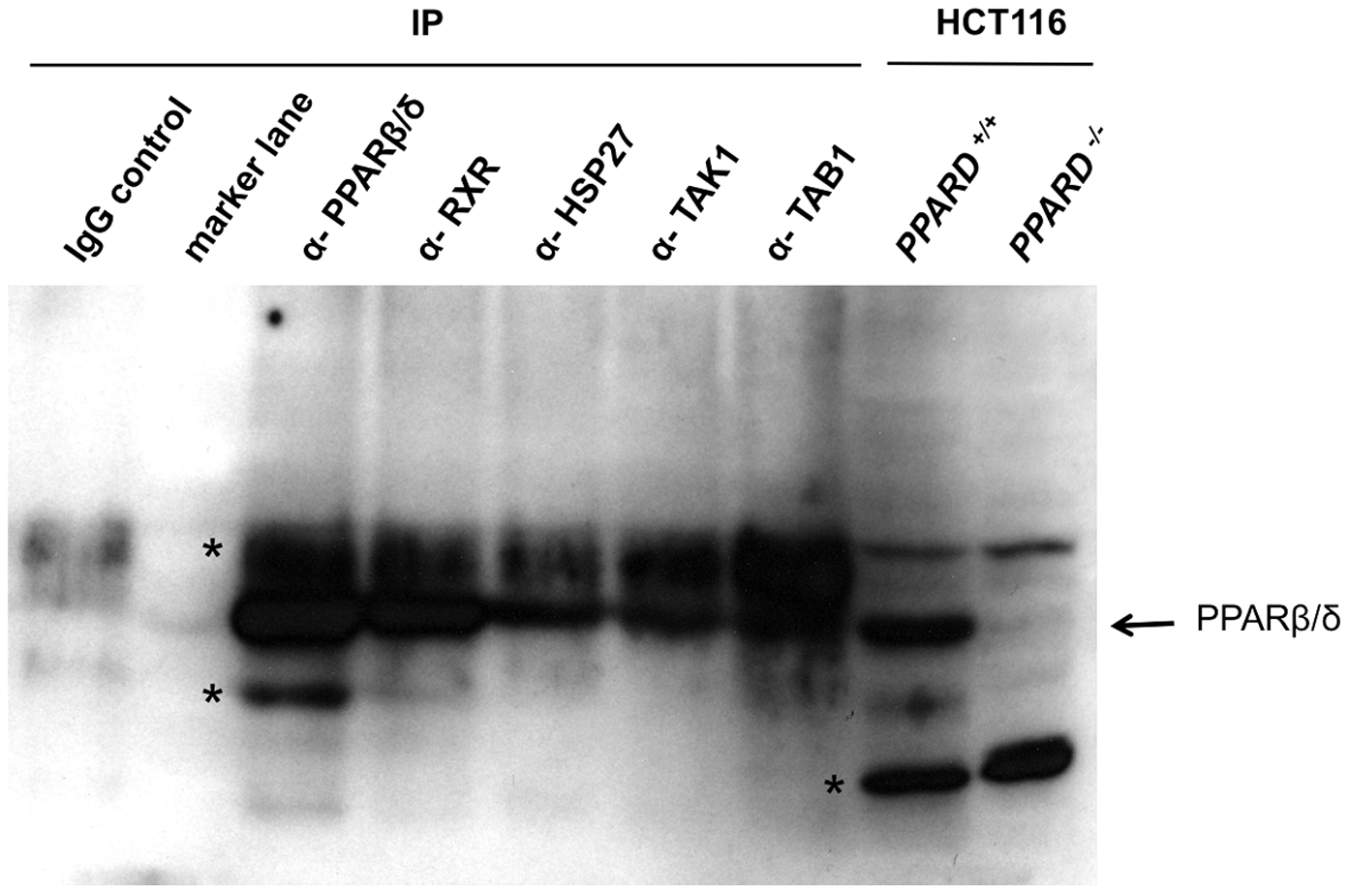
Interaction of endogenous PPARβ/δ with HSP27, TAK1 and TAB1. Untransfected HEK293T cells were treated with formaldehyde to stabilize protein interactions following the protocol for ChIP analyses. Cell extracts were prepared and immuneprecipitations were carried out with either irrelevant IgG or with antibodies against PPARβ/δ. RXR, HSP27, TAK1 or TAB1 (IP). Immunoblotting was performed with PPARβ/δ-specific antibodies. Antibodies against the established PPAR heterodimerization partner RXR were included as a positive control. The PPARβ/δ-HSP27 co-immunoprecipitation was abolished after pretreatment of the cell with HSP27 siRNA, confirming its specificity (not shown). The two rightmost lanes represent untreated extracts from HCT116 cells with intact (+/+) or disrupted (−/−) *PPARD* alleles [34] to allow for unambiguous identification of the PPARβ/δ band. *, non-specific band.

Importantly, interactions between PPARβ/δ and TAK1, TAB1 and HSP27 were not only detected between the overexpressed tagged proteins (Figures 6–8), but also between the endogenous proteins. Thus, as shown in Figure 9, TAK1, TAB1 and HSP27 were coprecipitated with PPARβ/δ in extracts from untransfected cells. Taken together with the fact that TAK1, TAB1 and HSP27 interact with each other, our finding are consistent with the formation of cytosolic signaling complex containing all four proteins, i.e. TAK1, TAB1, HSP27 and PPARβ/δ.

## Discussion

### Cytoplasmic Functions of PPARs

All known functions of PPARβ/δ are closely associated with chromatin regulation, and consistent with this role, PPARβ/δ localizes to the nucleus. However, there is evidence for an altered subcellular distribution of PPARs under certain circumstances. Thus, PPARα is constitutively cytoplasmic in differentiated macrophages [49] and a subpopulation of PPARα appears to be complexed with the chaperone HSP90 [50]. Furthermore, PPARs can shuttle between cytoplasm and nucleus [51], for example in the endothelial cell line EVC-304, where all three PPAR subtypes localize to the cytoplasm but translocate to the nucleus in response to 15-deoxy-prostaglandin J2 [52]. On the basis of immunostaining experiments, PPARβ/δ has been claimed to localize to the cytoplasm in different cell types [12], [53]–[55], although the limitations of this technique makes it difficult to draw clear-cut conclusions. Our cell fractionation studies with HEK293T cells (Figure 6F) also showed that a relatively large fraction of endogenous PPARβ/δ protein localizes to the cytoplasm, and thus support previous observations. Nevertheless, cytoplasmic functions of PPARβ/δ have not been described to date. In the present study, we report that PPARβ/δ directly targets and modulates the activity of cytoplasmic components of IL-1β signal transduction, which occurs independently of its known nuclear functions in transcriptional regulation.

### Modulation of a Subset of IL-1β Target Genes by PPARβ/δ

The combination of transcriptional profiling with siRNA-mediated interference identified a role for PPARβ/δ in maximizing the induction of a large subset of IL-1β target genes in HeLa cells, including pro-inflammatory effector genes, such as *IL6* and *IL8* (Figure 1A–E; Figure S2; Dataset S1). siRNA interference and gain-of-function strategies show that this effect of PPARβ/δ correlates with the extent of IL-1β induced chromatin binding of NFκB at these target genes (Figure 4A, B) and the phosphorylation and subsequent degradation of IκB (Figure 5B, C). However, this is not part of a global modulation of the NFκB response, since the IL-1β induction of a subset of target genes, including *BCL3* and *CXCL10*, are not significantly affected by PPARβ/δ depletion (Figure 1A; Figure S3; Dataset S2).

Although si-PPARD responsive and unresponsive IL-1β target genes are both regulated by NFκB (Figure 4A–D), they differ by several other criteria. First, the PPARβ/δ-regulated genes on average show a much stronger IL-1β response (up to 500-fold vs. maximum 32-fold for PPARβ/δ unresponsive genes; Figure 1B). Second, the kinetics of NFκB binding *in vivo* differ among the two sets of genes in that only the PPARβ/δ responsive genes show an increase in binding after 30 min of IL-1β stimulation (compare 30 and 45 min time points). These observations suggest that different transcription factor complexes assemble at the chromatin of both types of genes. Target gene selectivity is a well-known feature of NFκB driven transcriptional signaling. Different mechanisms have been proposed to explain this selectivity, including the gene-specific binding of distinct NFκB species, synergy between NFκB and gene-specific transcription factors and post-translational modifications of NFκB affecting specific co-regulator interactions [56], [57]. In this context it is noteworthy that PPARβ/δ depletion interferes with the phosphorylation of p65 at serine-536. It has previously been described that the phosphorylation of p65 at a different site in p65, Ser-468, is involved in specification of the transcriptional NFκB response [58]. It is therefore possible that the effect of PPARβ/δ on specific p65 kinase(s) is a determinant of its IL-1β target gene selectivity.

A previous study also described a role for PPARβ/δ in modulating the IL-1β response, but that study uncovered a functionally different mechanism compared to our findings. Thus, PPARβ/δ was reported to induce the secretion of the IL-1 receptor antagonist in dermal fibroblasts, which leads to an autocrine decrease in keratinocyte-induced IL-1β signaling pathways [17]. However, modulation of cytoplasmic IL-1β signaling intermediates by PPARβ/δ has not been reported to date.

We also found that the IL-1β-mediated target gene induction is not influenced by PPARβ/δ ligands (Figure S5). It is noteworthy that a ligand-independent function of PPARβ/δ as observed in the present study is not an unusual phenomenon, since this has also been observed with a subclass of direct PPARβ/δ target genes [3]. It is unlikely that this observation is due to endogenous agonists, since the antagonizing ligand ST247 had no effect on IL-1β-mediated target gene induction (Figure S5). It is possible that in this scenario PPARβ/δ has a non-receptor function, and that the level of PPARβ/δ, its post-translational modifications or the availability of cofactors control its activity. Alternatively, the function of PPARβ/δ described in the present study may not require any regulation, for example providing a platform for the assembly of a cytoplasmic multi-protein complex, as discussed further below.

### Regulation of TAK1/TAB1 Signaling by PPARβ/δ

A key event in the regulation of NFκB activity by PPARβ/δ appears to be its impact on TAK1/TAB1-mediated signaling (Figure 5A). This conclusion is based on the observations that PPARβ/δ physically interacts with cytoplasmic TAK1/TAB1 (Figures 6, 9) and that the siRNA-mediated depletion of PPARβ/δ interferes with the IL-1β-induced phosphorylation of TAK1 at Thr-187 (Figure 5B, C). This down-regulates the kinase activity of TAK1, as shown by the reduced phosphorylation of p38 at Thr-180/Tyr-182, of IκBα at Ser-32 and of p65 at Ser-536. The reduced phosphorylation of IκBα in turn inhibits its degradation.

We also performed in vitro TAK1 kinase assays using a MKK6-derived peptide as a substrate and immunoprecipitates form PPARβ/δ overexpressing HEK293T cells. However, these assays did not consistently show an induction upon PPARβ/δ overexpression (data not shown). This may be attributable to different reasons. It is possible that (i) the endogenous PPARβ/δ level is saturating, (ii) only a fraction of immuniprecipitated TAK1 is in a complex with PPARβ/δ, (iii) MKK6 may not be an appropriate substrate and/or (vi) the cell fractionation and immunoprecipitation conditions may alter TAK1 complex composition. At present, we can therefore not formally prove an effect of PPARβ/δ on TAK1 activity.

### Regulation of NFκB-driven Transcription by PPARβ/δ

Others have previously reported a putative role for PPARβ/δ in attenuating NFκB signaling. Thus, two studies have reported a physical interaction of PPARβ/δ with the p65 subunit of the NFκB dimer in cardiomyocytes and keratinocytes [11], [12], which we were able to reproduce in our experimental system. In addition, a PPARβ/δ agonist-mediated decrease in the steady level of p65 in an endothelial cell line [10] and a PPARβ/δ ligand-induced inhibition of IκB degradation has been observed in cardiomyocytes [13]. However, interpretation of these data is complicated by the fact that the PPARβ/δ dependence of the reported ligand effects is unclear, which is an essential issue in view of the off-target effects reported for PPARβ/δ agonists [59]–[61]. On the other hand, our data support the view that PPARβ/δ inhibits p65-driven transcription under specific circumstances, i.e., in the absence of IL-1β or TAK1/TAB1-induced signals (Figure 5F).

While the data discussed above point to an inhibitory effect of PPARβ/δ on nuclear NFκB, we have identified an independent cytoplasmic function of PPARβ/δ in the IL-1β triggered activation of NFκB (see scheme in Figure 5A). Thus, our data clearly show a stimulatory effect of PPARβ/δ overexpression on both IL-1β and TAK1/TAB1-induced signaling and the resulting NFκB activity (Fig. 5B–E). At present it is unclear how these opposite effects of PPARβ/δ are integrated within the NFκB signaling network. Based on the data in Figure 5F, one could hypothesize that the precise role of PPARβ/δ depends on specific conditions, such as the activation of signaling cascades (such as TAK1/TAB1) and the phosphorylation status of defined signaling components (such as p65). Further work will have to address these questions as well as potential cell type-specific effects of PPARβ/δ on NFκB signaling.

#### Interactions with HSP27 and formation of a multi-protein complex

Our data also indicate that HSP27 interacts with both PPARβ/δ and TAK1/TAB1 in the cytoplasm (Figures 8D–F and 9), and that both proteins independently enhance the IL-1β response of common target genes (Figure 8A, I). HSP27 is an ATP-independent molecular chaperone with functions in diverse biological processes including cell differentiation, proliferation and migration, tumor progression and metabolism [62]. In the context of IL-1β signaling, several previous reports on the functions of HSP27 are of particular relevance. First, phosphorylated HSP27 influences the stability of IL-1β induced mRNAs by promoting the proteolytic destruction of AUF1, an AU-rich element-binding protein that recruits different proteins to mRNA, including RNA degrading enzymes [63], [64]. However, actinomycin D treatment did not affect the PPARβ/δ effect on IL-1β target gene expression (Figure S10), indicating that mRNA stability is not modulated by the PPARβ/δ – HSP27 interaction.

Second, HSP27 interacts with TRAF6 to enhance its K63 ubiquitination, which in turn activates the kinase activity of IKK [33]. Our data suggest direct physical interactions of HSP27 with TAK1 and TAB1 (Figure 8E, F), pointing to a different mechanism. We also observe interactions of PPARβ/δ with TAK1, TAB1 and HSP27 (Figures 6A, B, D and 8D, E, 9), but not with TRAF6 (Figure 6C). Based on these findings, we propose a model where both PPARβ/δ and HSP27 interact both with each other and with TAK1/TAB1 to form a multi-protein complex and independently enhance TAK1/TAB1-mediated signaling (see Figure 5A). The function of PPARβ/δ in this context may be that of a scaffold protein that facilitates complex assembly, which would also be consistent the lack of regulation by PPARβ/δ ligands (Figure S5).

Taken together, our observations point an unexpected function for PPARβ/δ in modulating cytokine signaling mediated by its physical and functional interaction with cytoplasmic components of the IL-1β-triggered signal transduction cascade.

## Supporting Information

Figure S1 **Efficiency of siRNA-mediated silencing of PPARβ/δ.** HeLa cells were treated with control siRNA (si-con) or *PPARD*-directed siRNA (si-PPARD) and cell extracts were analyzed by RT-qPCR (panel A) or by immunoblotting using a PPARβ/δ-specific antibody (sc-74517; Santa Cruz) (panel B). We have previously shown that si-PPARD is specific for the β/δ subtype of PPAR proteins (Kaddatz et al., 2010).
(TIFF)

Figure S2 **Examples of IL-1β target genes affected by PPARβ/δ depletion (verification of microarray results; see Dataset S1).** HeLa cells were treated with control siRNA *(*si-con) or *PPARD*-directed siRNA (si-PPARD) followed by IL-1β (10 ng/ml) for 1 hr (see Figure S1 for knockdown efficiency). Expression patterns were determined by RT-qPCR. Values represent averages ±SD (*n* = 3). ***, **, *significant difference between si-con and si-PPARD-treated cells (p<0.001, *p*<0.01, *p*<0.05 by t-test).
(TIFF)

Figure S3 **Examples of IL-1β target genes not affected by PPARβ/δ depletion (verification of microarray results; see Dataset S1).** Experimental details and statistics as in Figure S2.
(TIFF)

Figure S4 **Examples of PPARβ/δ target genes derepressed by PPARβ/δ depletion but not affected by IL-1β (verification of microarray results; see Dataset S3).** Experimental details and statistics as in Figure S2.
(TIFF)

Figure S5 **PPARβ/δ ligands do not affect IL-1β-mediated target gene induction.** HeLa cells were treated with the agonist GW501516 (Sznaidman et al., 2003) or the inverse agonist ST247 (Naruhn et al., 2011) for 15 hrs followed by IL-1β (10 ng/ml) for 6 hr (see Figure S1 for knockdown efficiency). Expression patterns were determined by RT-qPCR. Statistics as in Figure S2.
(TIFF)

Figure S6 **Effect of individual** ***PPARD*** **-directed siRNAs on IL-1β induction of** ***IL6*** **.** HeLa cells were treated with control siRNA *(*si-con) or *PPARD*-directed siRNAs (si-PPARD) followed by IL-1β (10 ng/ml) for 6 hr. Expression levels of *PPARD* (A) and *IL6* (B) mRNAs were determined by RT-qPCR. Values represent averages ±SD (*n* = 3). ***, **, *significant difference between si-con and si-PPARD-treated cells (p<0.001, *p*<0.01, *p*<0.05 by t-test).
(TIFF)

Figure S7 **Effect of siRNA-mediated silencing of PPARβ/δ on TNFα-mediated target gene induction.** Recombinant human TNFα (20 ng/ml) was purchased from Sigma-Aldrich. Experimental details and statistics as in Figure S2.
(TIFF)

Figure S8 **Efficiency of siRNA-mediated silencing of HSP27.** HeLa cells were treated with control siRNA *(*si-con) or *HSP27*-directed siRNA (si-HSP27) and cell extracts were analyzed by RT-qPCR (panel A) or by immunoblotting using a HSP27-specific antibody (ADI-SPA-800; Stressgen) (panel B). Statistics as in Figure S2.
(TIFF)

Figure S9 **Examples of IL-1β target genes affected by HSP27 or PPARβ/δ depletion (verification of microarray results; see Dataset S4).** Experimental details and statistics as in Figure S2.
(TIFF)

Figure S10 **IL-1β target gene regulation by PPARβ/δ depletion is not affected by actinomycin D.** HeLa cells were treated with control siRNA *(*si-con) or *PPARD*-directed siRNA (si-PPARD) followed by IL-1β (20 ng/ml) for 90 min and actinomycin D (5 µg/ml) for 30 min. Expression of *IL6* mRNA was determined by RT-qPCR. Values represent averages ±SD (*n* = 3). Statistics as in Figure S2.
(TIFF)

Table S1 **Primers for site-directed mutagenesis and PCR cloning of the PPARβ/δ constructs.**
(PDF)

Table S2 **siRNA sequences.**
(PDF)

Table S3 **Primers for RT-qPCR.**
(PDF)

Table S4 **Primers for ChIP assays.**
(PDF)

Dataset S1 **Microarray analysis of HeLa cells treated with IL-1β in the presence of si-PPARD or control siRNA: complete list of genes with IL-1β response (≥2-fold) modulated ≥1.8-fold by si-PPARD.**
(XLS)

Dataset S2 **Microarray analysis of HeLa cells treated with IL-1β in the presence of si-PPARD or control siRNA: complete list of genes with IL-1β response (≥2-fold) unaffected by si-PPARD (≤1.4-fold).**
(XLS)

Dataset S3 **Microarray analysis of HeLa cells treated with si-PPARD in the presence of IL-1β: complete list or all PPARβ/δ target genes (≥1.8-fold).**
(XLS)

Dataset S4 **Microarray analysis of HeLa cells treated with** ***HSP27, PPARD*** **or control siRNA: complete list of genes inhibited by at least one siRNA (>1.5-fold).**
(XLS)
