# Supplementary figures and images for: Regulation of TAK1/TAB1-Mediated IL-1β Signaling by Cytoplasmic PPARβ/δ
Source: PLoS One. 2013 Apr 30;8(4):e63011. doi: 10.1371/journal.pone.0063011 (PMC3639976; doi:10.1371/journal.pone.0063011)

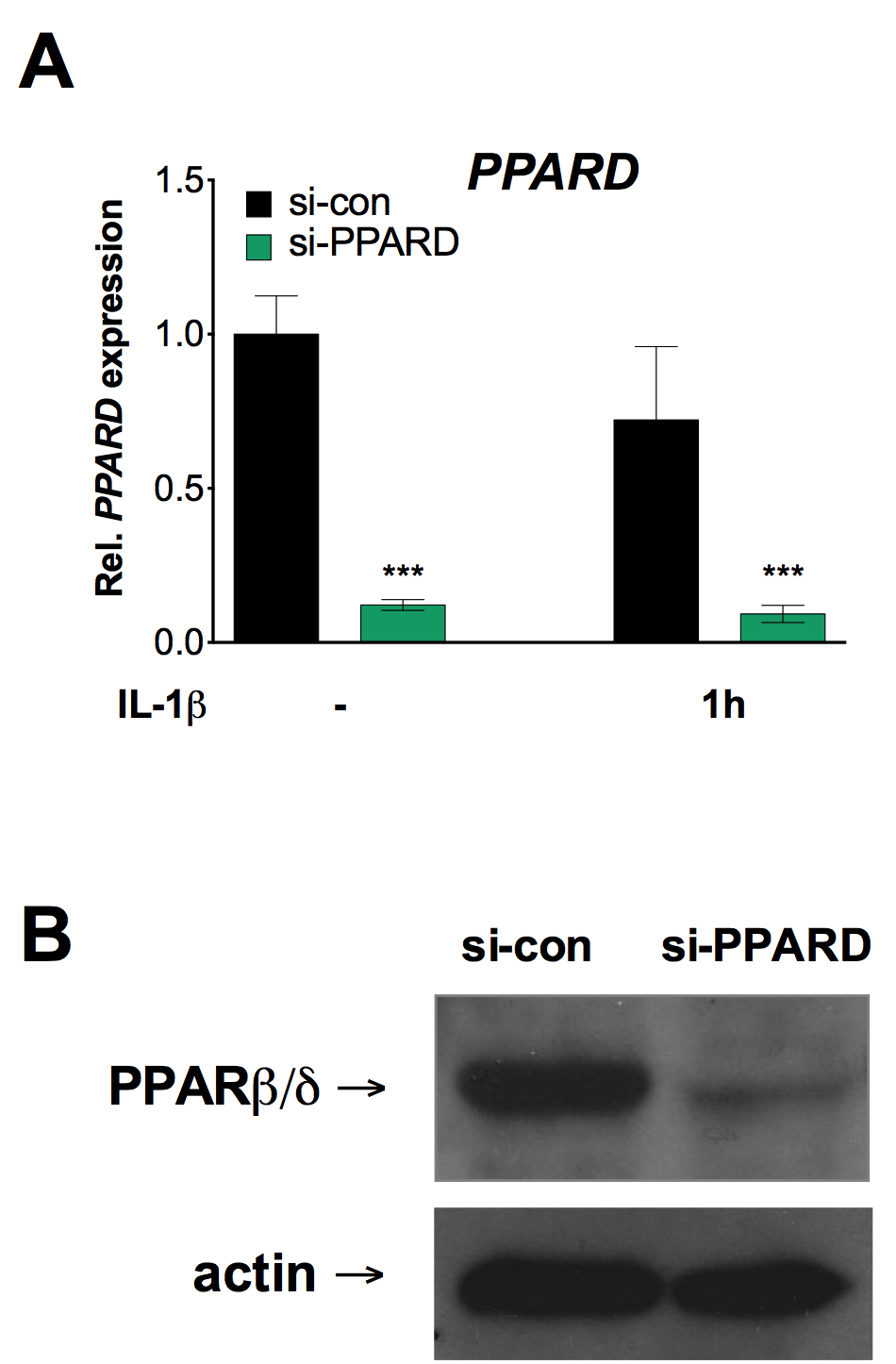

Supplement: Figure S1 — Efficiency of siRNA-mediated silencing of PPARβ/δ. HeLa cells were treated with control siRNA (si-con) or PPARD-directed siRNA (si-PPARD) and cell extracts were analyzed by RT-qPCR (panel A) or by immunoblotting using a PPARβ/δ-specific antibody (sc-74517; Santa Cruz) (panel B). We have previously shown that si-PPARD is specific for the β/δ subtype of PPAR proteins (Kaddatz et al., 2010). (TIFF) [file pone.0063011.s001.tiff]

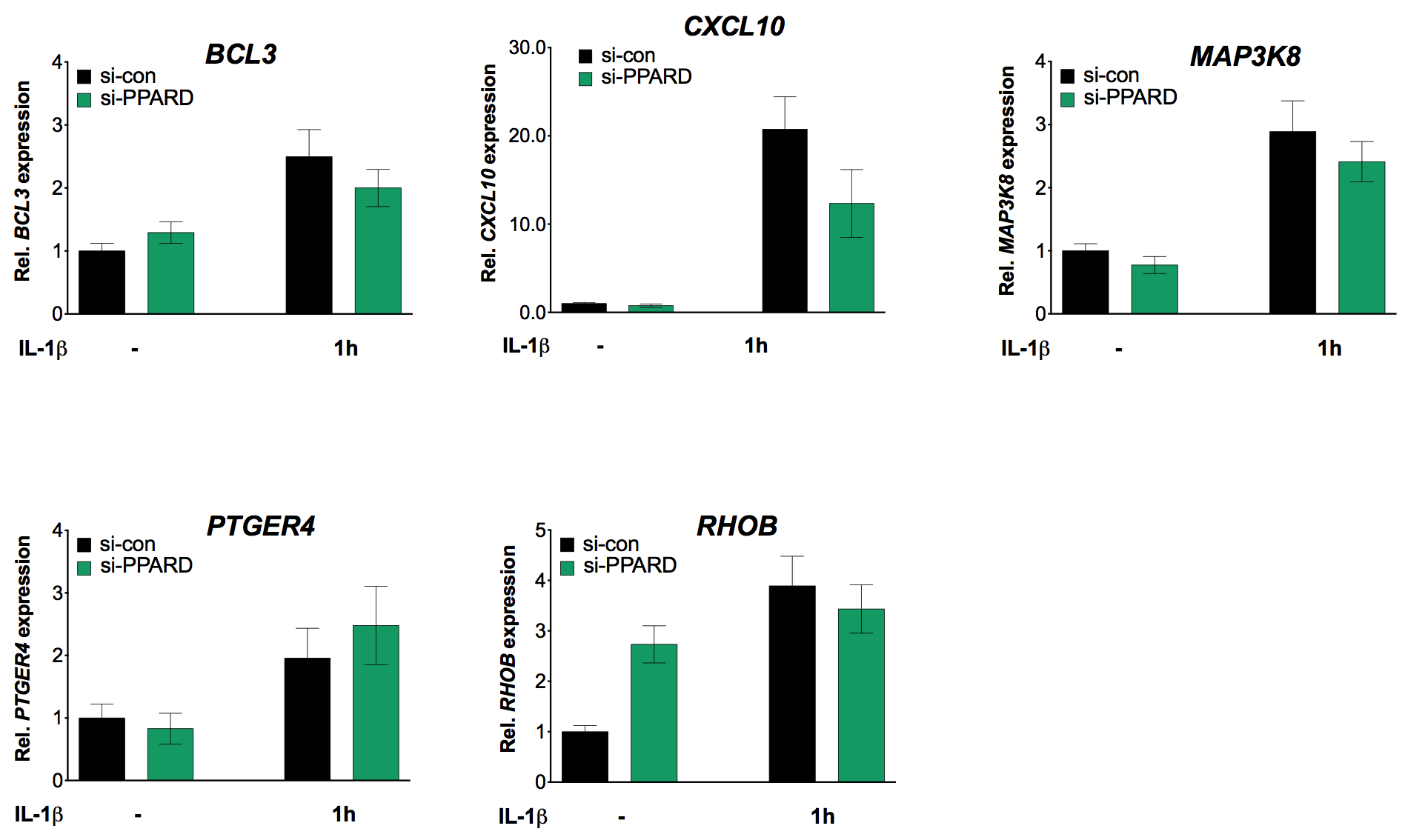

Supplement: Figure S2 — Examples of IL-1β target genes affected by PPARβ/δ depletion (verification of microarray results; see Dataset S1). HeLa cells were treated with control siRNA (si-con) or PPARD-directed siRNA (si-PPARD) followed by IL-1β (10 ng/ml) for 1 hr (see Figure S1 for knockdown efficiency). Expression patterns were determined by RT-qPCR. Values represent averages ±SD (n = 3). ***, **, *significant difference between si-con and si-PPARD-treated cells (p<0.001, p<0.01, p<0.05 by t-test). (TIFF) [file pone.0063011.s002.tiff]

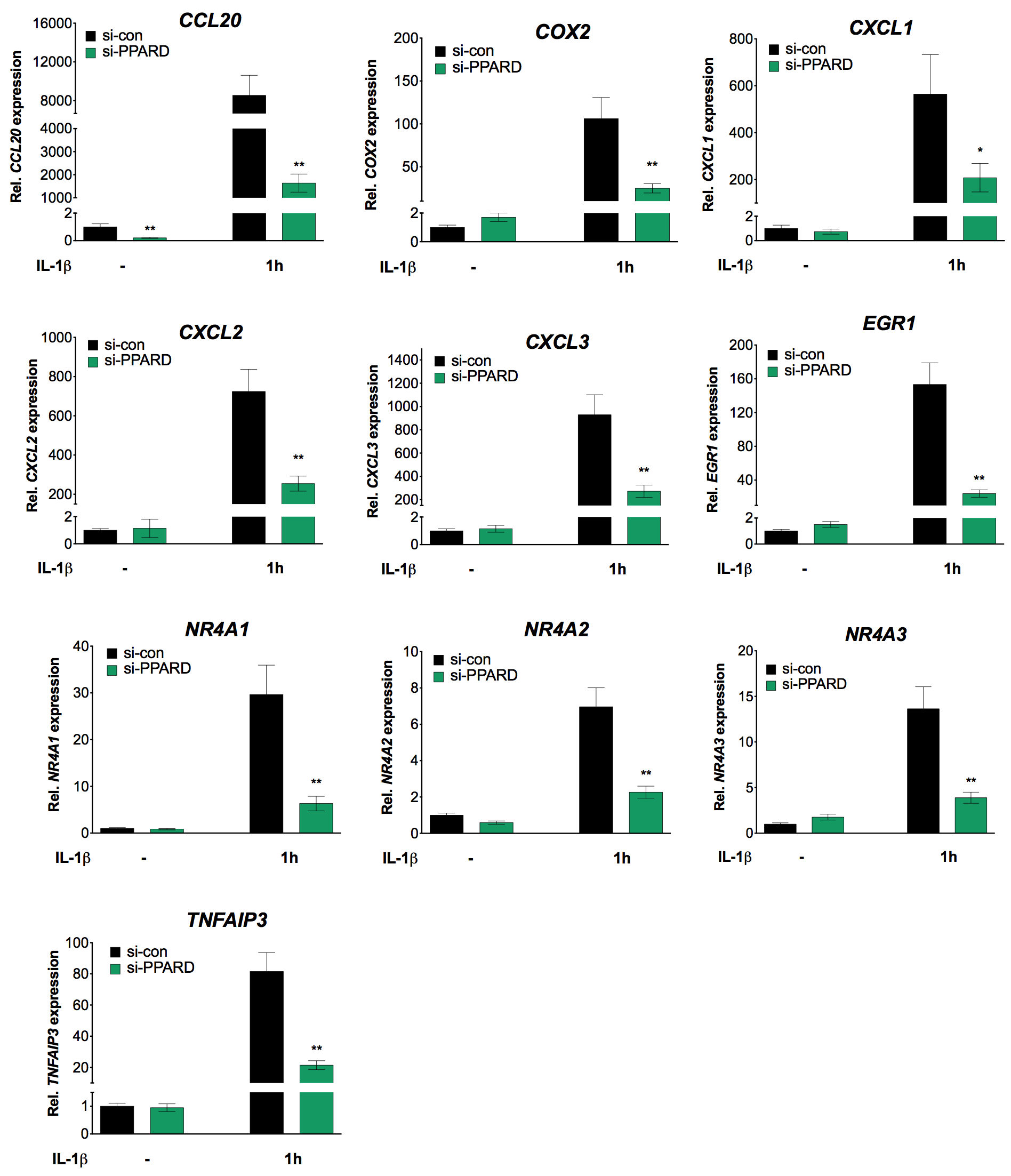

Supplement: Figure S3 — Examples of IL-1β target genes not affected by PPARβ/δ depletion (verification of microarray results; see Dataset S1). Experimental details and statistics as in Figure S2. (TIFF) [file pone.0063011.s003.tiff]

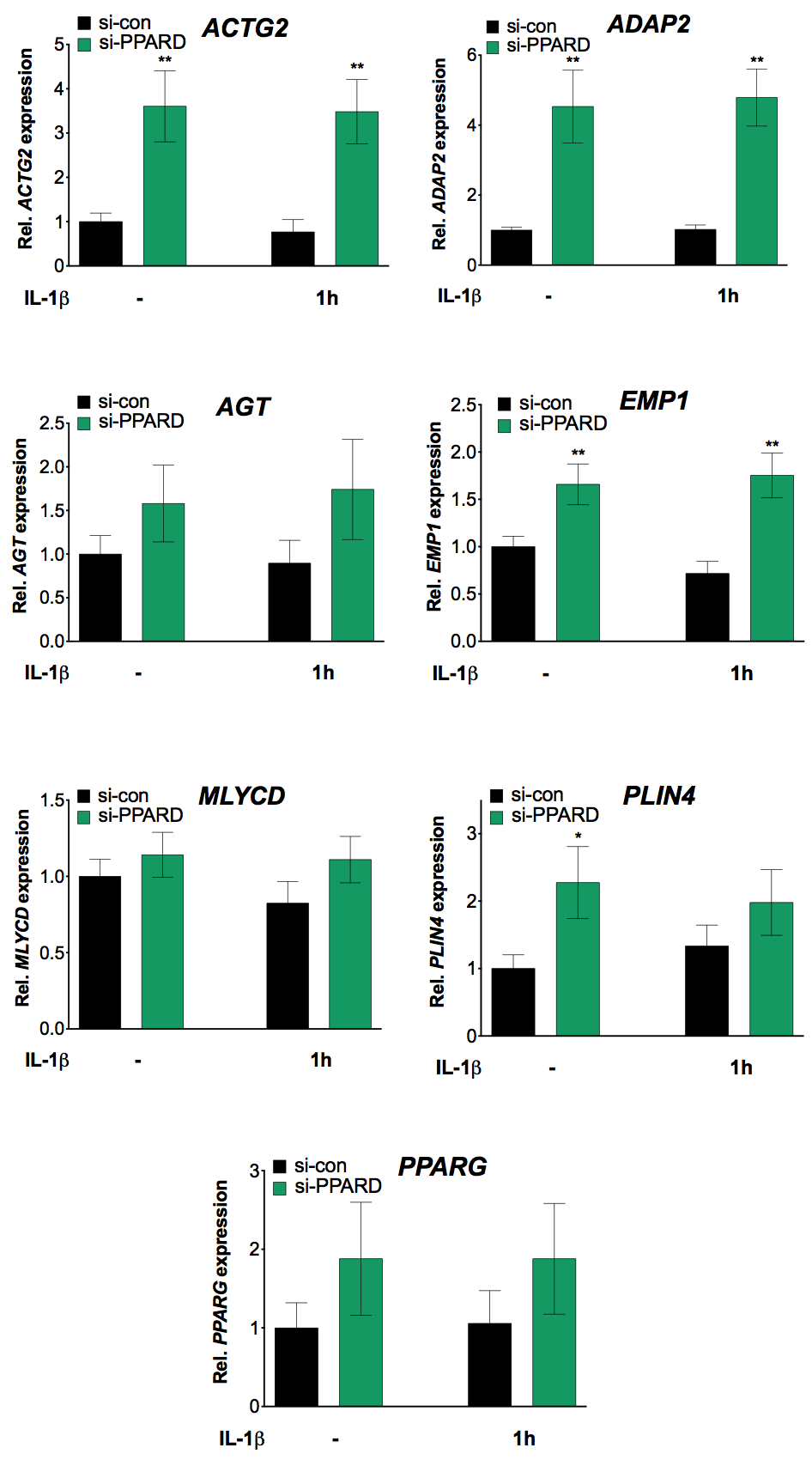

Supplement: Figure S4 — Examples of PPARβ/δ target genes derepressed by PPARβ/δ depletion but not affected by IL-1β (verification of microarray results; see Dataset S3). Experimental details and statistics as in Figure S2. (TIFF) [file pone.0063011.s004.tiff]

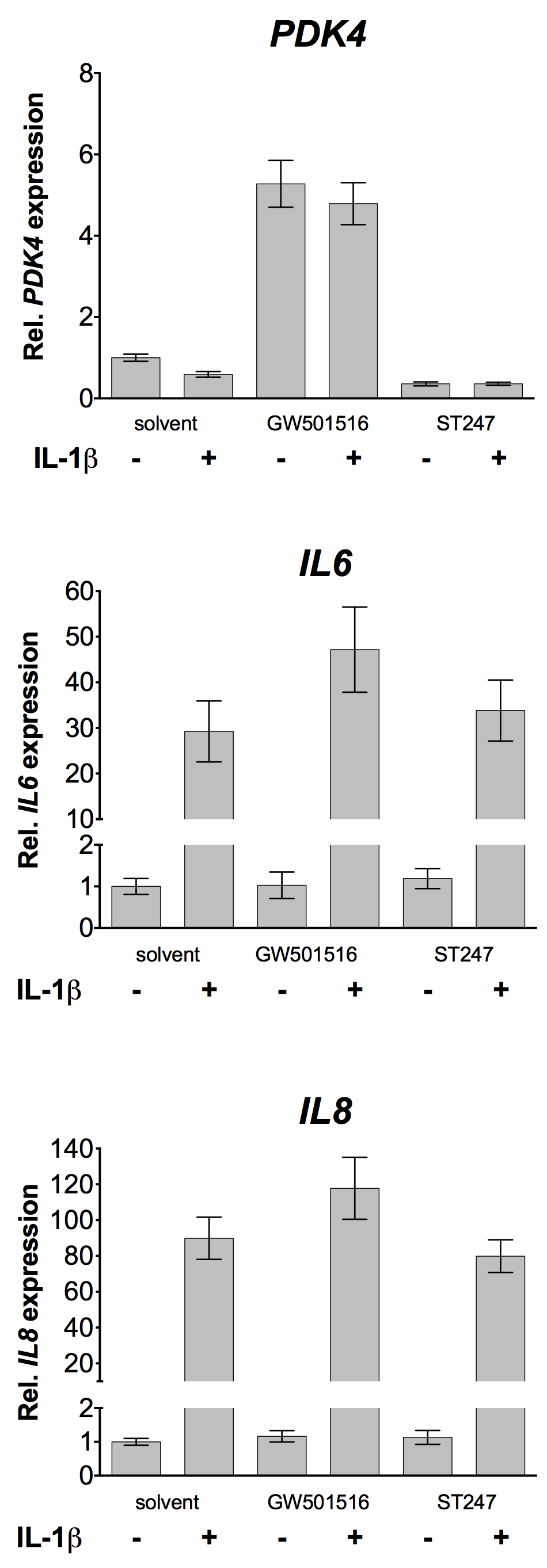

Supplement: Figure S5 — PPARβ/δ ligands do not affect IL-1β-mediated target gene induction. HeLa cells were treated with the agonist GW501516 (Sznaidman et al., 2003) or the inverse agonist ST247 (Naruhn et al., 2011) for 15 hrs followed by IL-1β (10 ng/ml) for 6 hr (see Figure S1 for knockdown efficiency). Expression patterns were determined by RT-qPCR. Statistics as in Figure S2. (TIFF) [file pone.0063011.s005.tiff]

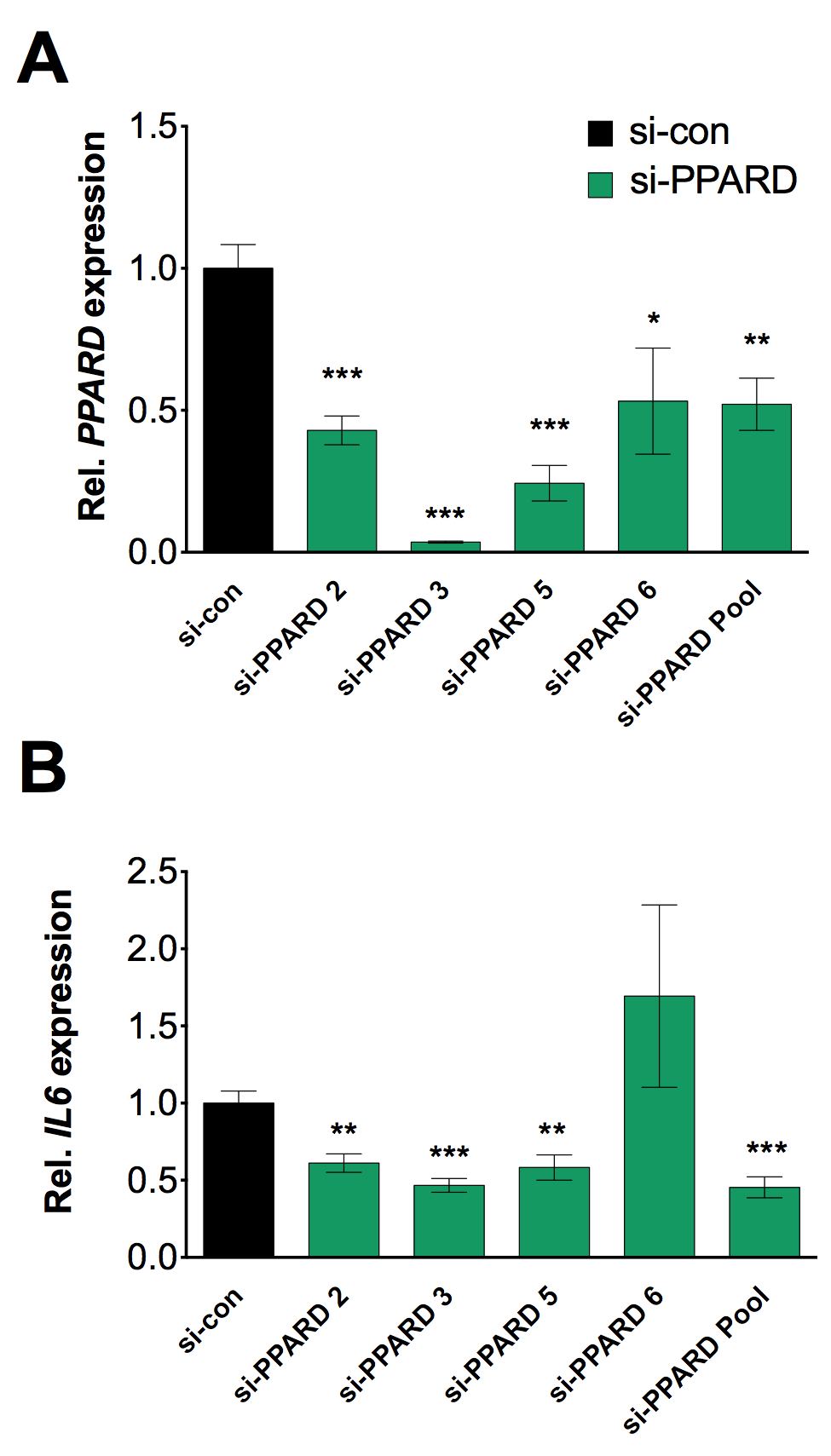

Supplement: Figure S6 — Effect of individual PPARD -directed siRNAs on IL-1β induction of IL6 . HeLa cells were treated with control siRNA (si-con) or PPARD-directed siRNAs (si-PPARD) followed by IL-1β (10 ng/ml) for 6 hr. Expression levels of PPARD (A) and IL6 (B) mRNAs were determined by RT-qPCR. Values represent averages ±SD (n = 3). ***, **, *significant difference between si-con and si-PPARD-treated cells (p<0.001, p<0.01, p<0.05 by t-test). (TIFF) [file pone.0063011.s006.tiff]

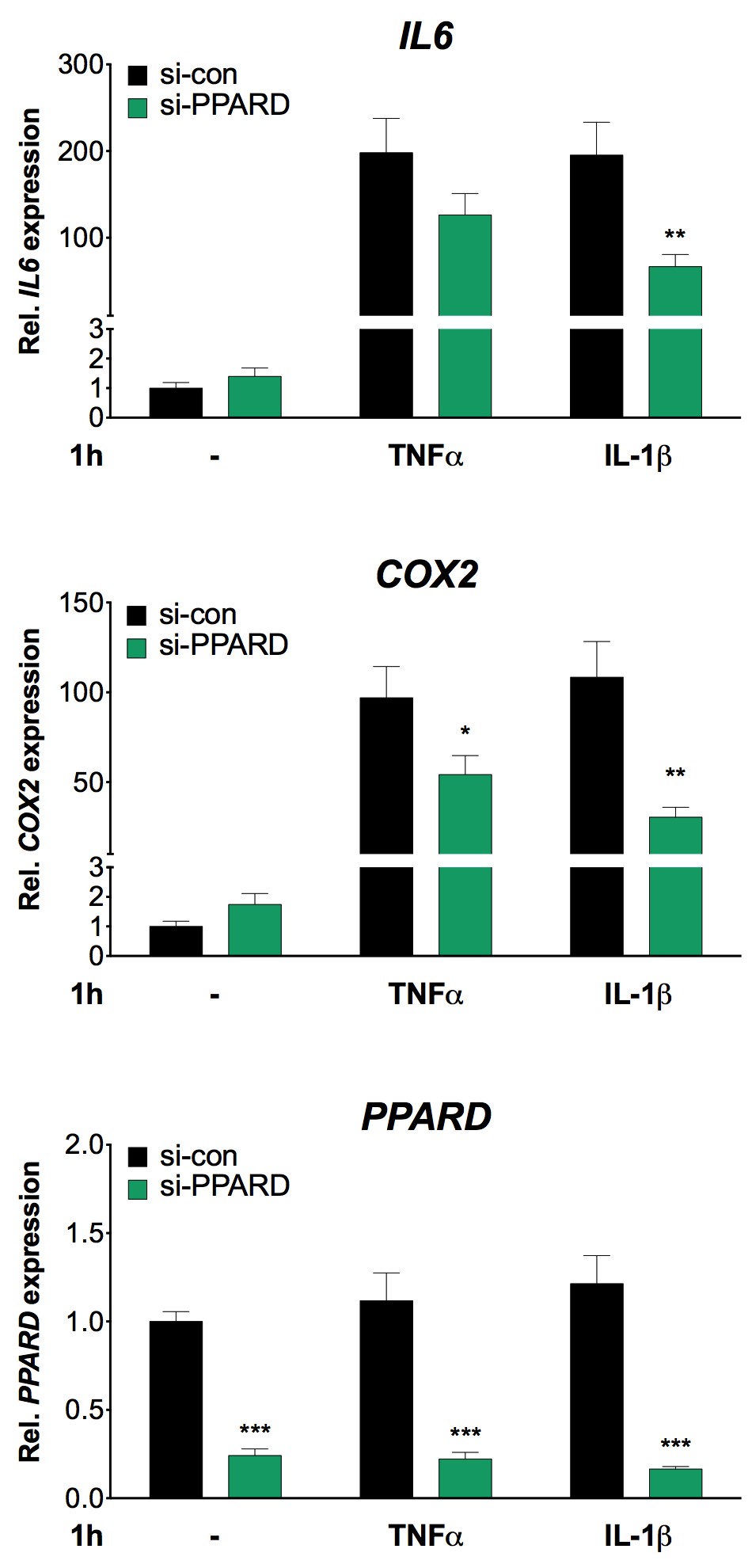

Supplement: Figure S7 — Effect of siRNA-mediated silencing of PPARβ/δ on TNFα-mediated target gene induction. Recombinant human TNFα (20 ng/ml) was purchased from Sigma-Aldrich. Experimental details and statistics as in Figure S2. (TIFF) [file pone.0063011.s007.tif]

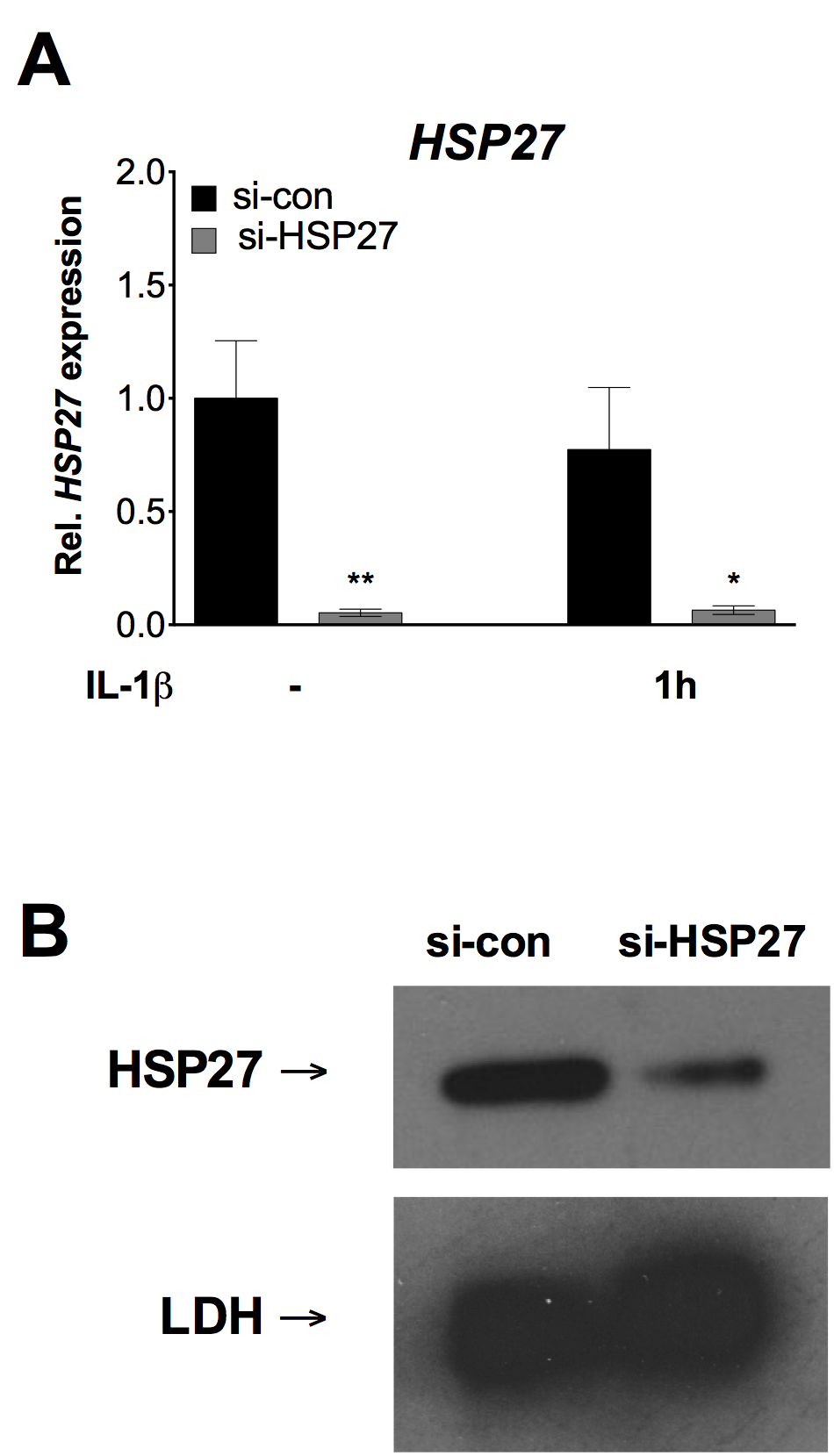

Supplement: Figure S8 — Efficiency of siRNA-mediated silencing of HSP27. HeLa cells were treated with control siRNA (si-con) or HSP27-directed siRNA (si-HSP27) and cell extracts were analyzed by RT-qPCR (panel A) or by immunoblotting using a HSP27-specific antibody (ADI-SPA-800; Stressgen) (panel B). Statistics as in Figure S2. (TIFF) [file pone.0063011.s008.tif]

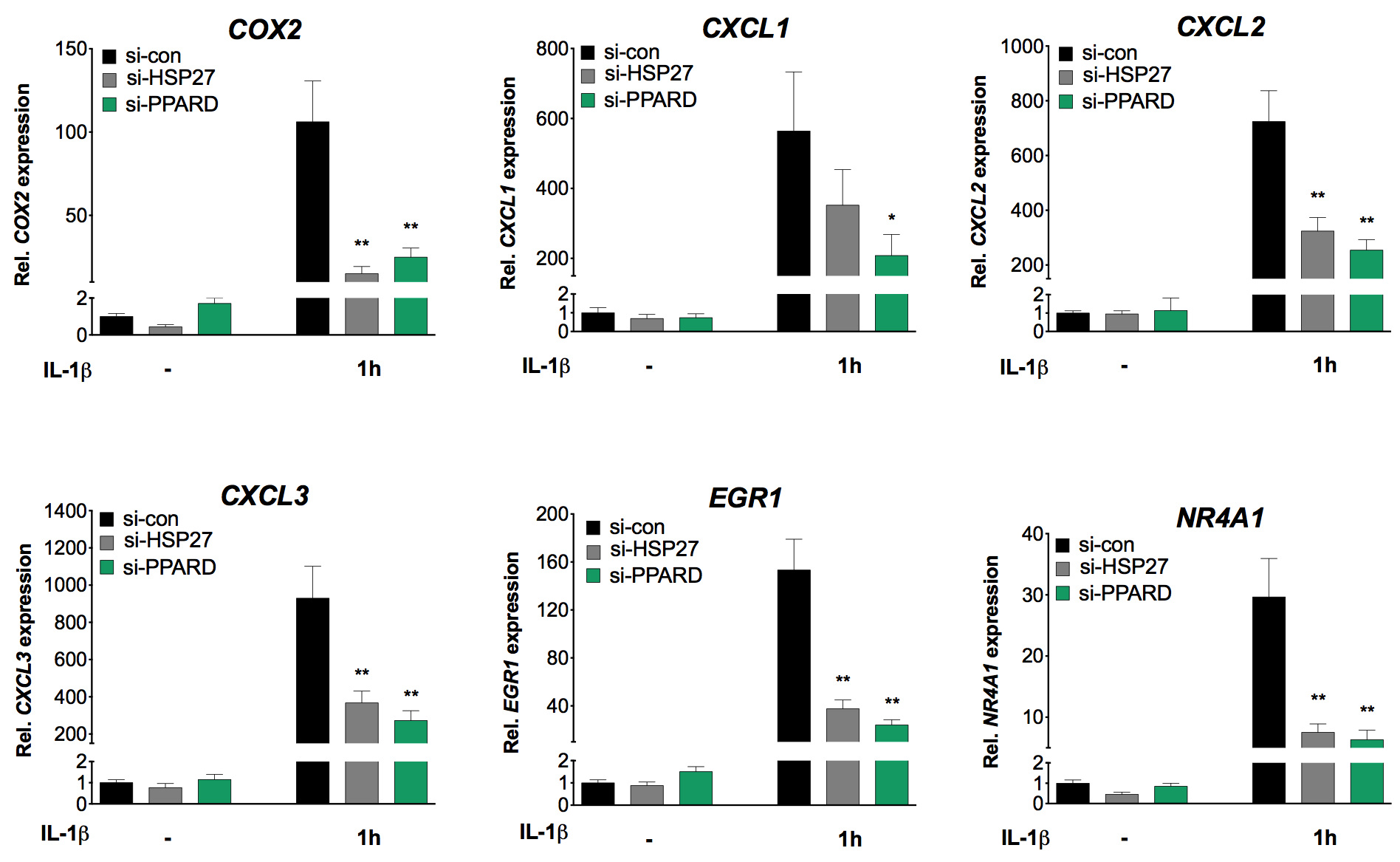

Supplement: Figure S9 — Examples of IL-1β target genes affected by HSP27 or PPARβ/δ depletion (verification of microarray results; see Dataset S4). Experimental details and statistics as in Figure S2. (TIFF) [file pone.0063011.s009.tif]

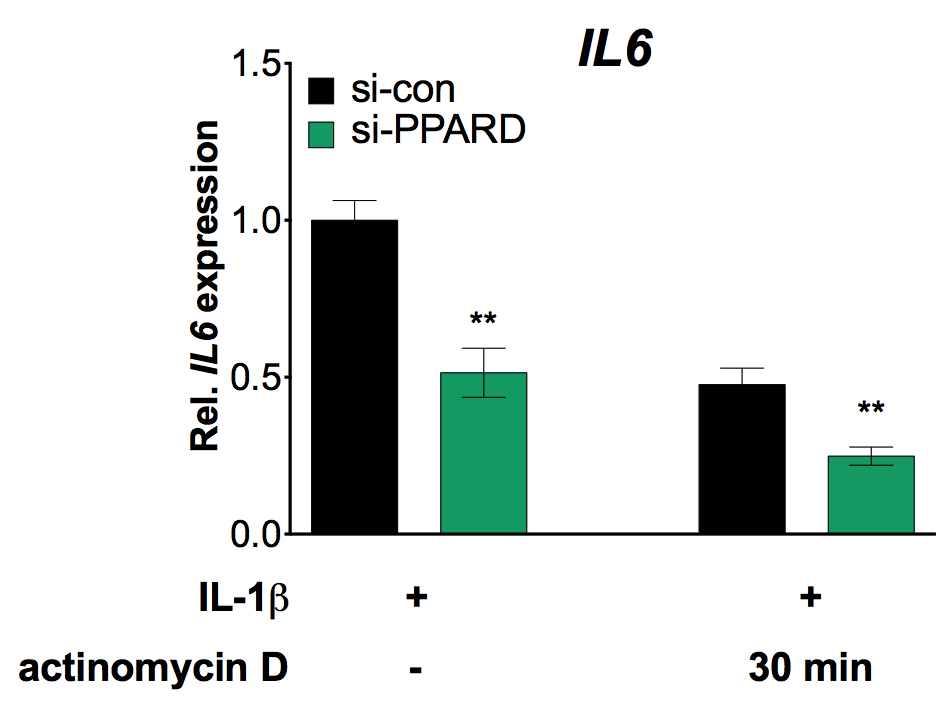

Supplement: Figure S10 — IL-1β target gene regulation by PPARβ/δ depletion is not affected by actinomycin D. HeLa cells were treated with control siRNA (si-con) or PPARD-directed siRNA (si-PPARD) followed by IL-1β (20 ng/ml) for 90 min and actinomycin D (5 µg/ml) for 30 min. Expression of IL6 mRNA was determined by RT-qPCR. Values represent averages ±SD (n = 3). Statistics as in Figure S2. (TIFF) [file pone.0063011.s010.tif]
