# Supplementary material for: Regulation of TAK1/TAB1-Mediated IL-1β Signaling by Cytoplasmic PPARβ/δ
Source: PLoS One. 2013 Apr 30;8(4):e63011. doi: 10.1371/journal.pone.0063011 (PMC3639976; doi:10.1371/journal.pone.0063011)
Supplement: Table S1 — Primers for site-directed mutagenesis and PCR cloning of the PPARβ/δ constructs. (PDF) [file pone.0063011.s011.pdf]

Table S1: Primers for site-directed mutagenesis and PCR cloning of the PPARβ/δ constructs

3xFLAG PPARβ/δ 4-165:

5’ –TGACTGCCAGCGAGGGGTGCTAGCACAA

3xFLAG PPARβ/δ 166-440:

5’ -ATGGATCCTCAGCACAACCCCCAGC

5’ -CATCTCGAGTTAGTACATGTCCTTG
