# Supplementary material for: Regulation of TAK1/TAB1-Mediated IL-1β Signaling by Cytoplasmic PPARβ/δ
Source: PLoS One. 2013 Apr 30;8(4):e63011. doi: 10.1371/journal.pone.0063011 (PMC3639976; doi:10.1371/journal.pone.0063011)
Supplement: Table S2 — siRNA sequences. (PDF) [file pone.0063011.s012.pdf]

Table S2: siRNA sequences

Hs_PPARD_2:	5' – CAG ACU GAC GAA ACU UUA A
Hs_PPARD_3:	5' – GUG AUA UCA UUG AGC CUA A
Hs_PPARD_5:	5' – GCG GAU CAA GAA GAC CGA A
Hs_PPARD_6:	5' – GGU UAC CCU UCU CAA GUA U
HSP27_01:	5' – CCG AUG AGA CUG CCG CCA A
HSP27_03:	5' – GGC AGG ACG AGC AUG GCU A
HSP27_04:	5' – CCG GAG GAG UGG UCG CAG U
HSP27_05:	5' – CAA GUU UCC UCC UCC CUG U
control siRNA:	5' – CAG UCG CGU UUG CGA CUG G
