# Supplementary material for: Regulation of TAK1/TAB1-Mediated IL-1β Signaling by Cytoplasmic PPARβ/δ
Source: PLoS One. 2013 Apr 30;8(4):e63011. doi: 10.1371/journal.pone.0063011 (PMC3639976; doi:10.1371/journal.pone.0063011)
Supplement: Table S3 — Primers for RT-qPCR. (PDF) [file pone.0063011.s013.pdf]

Table S3: Primers for RT-qPCR

L27_fw:	5' – AAA GCC GTC ATC GTG AAG AAC
L27_rv:	5' – GCT GTC ACT TTC CGG GGA TAG
PPARD_fw:	5' – TCA TTG CGG CCA TCA TTC TGT GTG
PPARD_rv:	5' – TTC GGT CTT CTT GAT CCG CTG CAT
HSP27_fw:	5' – TCC CTG GAT GTC AAC CAC TT
HSP27_rv:	5' – GAT GTA GCC ATG CTC GTC CT
ANGPTL4_fw:	5' – GAT GGC TCA GTG GAC TTC AAC C
ANGTPL4_rv:	5' – CCC GTG ATG CTA TGC ACC TTC
SOCS3_fw:	5' – ATC CTG GTG ACA TGC TCC TC
SOCS3_rv:	5' – CAA ATG TTG CTT CCC CCT TA
IL6_fw:	5' – CAG GAG CCC AGC TAT GAA CT
IL6_rv:	5' – AGC AGG CAA CAC CAG GAG
IL8_fw:	5' – ATG ACT TCC AAG CTG GCC GTG GCT
IL8_rv:	5' – TCT CAG CCC TCT TCA AAA ACT TCT C
COX2_fw:	5' – TGA ATC ATT TGA AGA ACT TAC AG
COX2_rv:	5' – CAT CTG GCC GAG GCT TTT C
CCL20_fw:	5' – GCT GCT TTG ATG TCA GTG CT
CCL20_rv:	5' – GCA GTC AAA GTT GCT TGC TTC
CXCL1_fw:	5' – TCC TGC ATC CCC CAT AGT TA
CXCL1_rv:	5' – CTT CAG GAA CAG CCA CCA GT
CXCL2_fw:	5' – CCC ATG GTT AAG AAA ATC ATC G
CXCL2_rv:	5' – CTT CAG GAA CAG CCA CCA AT
CXCL3_fw:	5' – AAA ATC ATC GAA AAG ATA CTG AAC AA
CXCL3_rv:	5' – GGT AAG GGC AGG GAC CAC
EGR1_fw:	5' – AGC CCT ACG AGC ACC TGA C
EGR1_rv:	5' – GGT TTG GCT GGG GTA ACT G
NR4A1_fw:	5' – CCA CTG CCT CCT TCA ACC
NR4A1_rv:	5' – GGC TTG GAT ACA GGG CAT C
NR4A2_fw:	5' – TGA AGA GAG ACG CGG AGA AC
NR4A2_rv:	5' – AAA GCA ATG GGG AGT CCA G
NR4A3_fw:	5' – ACA CCC AGA GAT CTT GAT TAT TCC
NR4A3_rv:	5' – GTA GAA TTG TTG CAC ATG CTC AG

TNFAIP3_fw:	5' – TGC ACA CTG TGT TTC ATC GAG
TNFAIP3_rv:	5' – ACG CTG TGG GAC TGA CTT TC
BCL3_fw:	5' – CGA CAT CTA CAA CAA CCT ACG G
BCL3_rv:	5' – CCA CAG ACG GTA ATG TGG TG
CXCL10_fw:	5' – GAA AGC AGT TAG CAA GGA AAG GT
CXCL10_rv:	5' – GAC ATA TAC TCC ATG TAG GGA AGT GA
MAP3K8_fw:	5' – CGC AAG AGG CTG CTG AGT A
MAP3K8_rv:	5' – TTC CTG TGC ACG AAG AAT CA
PTGER4_fw:	5' – CTC CCT GGT GGT GCT CAT
PTGER4_rv:	5' – GGC TGA TAT AAC TGG TTG ACG A
RHOB_fw:	5' – CCC CTG AGC ATG CTT TTC T
RHOB_rv:	5' – CGA GGG GAG TCG AAC AGA C
SAT1_fw:	5' – CCT ATG ACC CGT GGA TTG GC
SAT1_rv:	5' – TGC AAC CTG GCT TAG ATT CTT C
ACTG2_fw:	5' – CTC TCA AAT ACC CCA TTG AAC AC
ACTG2_rv:	5' – AGG AGT GGT GCC AGA TCT TC
ADAP2_fw:	5' – CCA CCG TAA CTT CCC TGA CAT
ADAP2_rv:	5' – AAA CTC CAC AAT ACT GTC GTC C
AGT_fw:	5' – TCA ACA CCT ACG TCC ACT TCC
AGT_rv:	5' – GCT GTT GTC CAC CCA GAA CT
EMP1_fw:	5' – TCC AGA AGA GCG GAC CAG
EMP1_rv:	5' – ACT TGT GAG CAA AGA GAG TTC TGA
PDK4_fw:	5' – TTA TAC ATA CTC CAC TGC ACC A
PDK4_rv:	5' – ATA GAC TCA GAA GAC AAA GCC T
PPARG_fw:	5' – TGC ACT GGA ATT AGA TGA CAG C
PPARG_rv:	5' – TCC GTG ACA ATC TGT CTG AGG
PLIN4_fw:	5' – GGA GCT GCA ACC TTC GGA AA
PLIN4_rv:	5' – GGA CCA CTC CCT TAG CCA C
