# Supplementary material for: Regulation of TAK1/TAB1-Mediated IL-1β Signaling by Cytoplasmic PPARβ/δ
Source: PLoS One. 2013 Apr 30;8(4):e63011. doi: 10.1371/journal.pone.0063011 (PMC3639976; doi:10.1371/journal.pone.0063011)
Supplement: Table S4 — Primers for ChIP assays. (PDF) [file pone.0063011.s014.pdf]

Table S4: Primers for ChIP assays

control_fw	5’ – GTA TGT GTA CTG GGG GGA C
control_rv	5’ – CAC ATG GCT CTT TTC GTT CC
ANGPTL4-PPRE_fw	5’ – CGC AGA GTG ACC AGG AAG AC
ANGPTL4-PPRE_rv	5’ – CCT TAC TGG ATG GGA GGA AAG
IL6-NFκB_fw	5’ – CCC TCA CCC TCC AACAAA G
IL6-NFκB_rv	5’ – TTG TGG AGA AGG AGT TCA TAG C
IL8-NFκB_fw	5’ – CCA AAT TGT GGA GCT TCA GT
IL8-NFκB_rv	5’ – TTC CTT CCG GTG GTT TCT TC
BCL3-NFκB_fw	5’ – CCG TGT CTC TTG CTA TCT CTC
BCL3-NFκB_rv	5’ – GCG CTC CAC TGA TTG TGT C
CXCL10-NFκB_fw	5’ – GAG TCT GCA ACA TGG GAC TTC
CXCL10-NFκB_rv	5’ – AAA GCC ATT TTC CCT CCC TA
